# Salt stress-induced remodeling of sugar transport: a role for promoter alleles of *SWEET13*

**DOI:** 10.1038/s41598-025-90432-2

**Published:** 2025-03-04

**Authors:** Eman Abuslima, Adnan Kanbar, Ahmed Ismail, Manish L. Raorane, Elisabeth Eiche, Islam El-Sharkawy, Björn H. Junker, Michael Riemann, Peter Nick

**Affiliations:** 1 Molecular Cell Biology, Joseph Kölreuter Institute for Plant Sciences, Karlsruhe Institute of Technology, Fritz-Haber-Weg 4, 76131 Karlsruhe, Germany; 2 Department of Botany and Microbiology, Faculty of Science, Suez Canal University, Ismailia, 41522 Egypt; 3 Department of Horticulture, Faculty of Agriculture, Damanhour University, Damanhour, 22516 Egypt; 4 Department of Botany and Plant Sciences, University of California Riverside, Riverside, CA 92521 USA; 5 Institute of Pharmacy, Martin-Luther-University, Halle-Wittenberg, Hoher Weg 8, 06120 Halle (Saale), Germany; 6 Institute of Applied Geosciences, Karlsruhe Institute of Technology, 76131 Karlsruhe, Germany; 7 Laboratory for Environmental and Raw Materials Analysis (LERA), Karlsruhe Institute of Technology, 76131 Karlsruhe, Germany; 8 Center for Viticulture and Small Fruit Research, College of Agriculture and Food Sciences, Florida A&M University, Tallahassee, FL 32308 USA; 9 Department of Field Crops, Faculty of Agriculture, University of Damascus, PO Box 30621, Damascus, Syria

**Keywords:** Salt stress, Sorghum, Sucrose, Sugar transport, SUTs, SWEETs, Plant molecular biology, Plant stress responses, Plant sciences

## Abstract

Sucrose, the primary carbon form synthesized by photosynthesis, is transported via the phloem for proper plant development and productivity. However, long-distance sucrose transport can become unbalanced under adverse environmental conditions. Therefore, we highlight the influence of salt stress on sugar partitioning in source versus sink tissues in sorghum under generative development including the role of stress induced sucrose transporter expression. The two sorghum genotypes displayed different responses to salinity in terms of resource allocation, in Della sugar was translocated to the stem and roots, whereas in Razinieh sugars were directed towards the grains. In Della, the unloading of sucrose in the roots was associated with increased expression levels of *SbSUT6* and *SbSWEET6*, while in the internodes, sucrose unloading correlated with elevated levels of *SbSWEET13* and the ABA-dependent transcription factor *SbbZIP-TF-TRAB1*. Conversely, in Razinieh, the expression of *SbSUT2* in the flag internodes was linked to enhanced panicle development. In addition, a differential activation of *SbSWEET13* and *SbSUT6* promoters by ABA and MeJA was elucidated using dual-luciferase reporter assay in sorghum protoplasts. Finally, we arrive at a model where dynamic remodeling of sugar transport during generative development is crucial for the response to salt stress, and more manifested in sink tissues.

**Supplementary Information:**

The online version contains supplementary material available at 10.1038/s41598-025-90432-2.

## Introduction

The global population is expected to skyrocket by 34% till 2050, which means that crop productivity, especially that of cereals, needs to increase by 43% to meet the growing demand^1^. The impact of climate change renders it progressively difficult to meet this goal, food security is threatened by extreme weather events, such as extreme drought, excessive and untimely heat, wildfires, or sudden floods, but also by rising sea levels that affect coastal areas, where frequently the most productive areas are located^2,3^. Therefore, yield increases will only be sustainable, if accompanied by improved climate resilience for instance, through introgression of germplasm tolerant to abiotic stresses^4,5^. While there is a public awareness for the impact of heat and drought stress, soil salinity, being one of the major abiotic stresses aggravated by climate change has not attracted the degree of attention that would correspond to its massive impact on global agriculture. Currently, 831 million hectares of worldwide lands are affected by salinity, and this toll is predicted to increase by an additional 50% by 2050. As a consequence of soil salinity, freshwater becomes limited, accentuating the competition for drinking water, and often forcing farmers to use brackish low-quality water for irrigation, deteriorating the situation even further^6^. Overcoming this vicious circle will not only depend on improved practices of land management, but also on the breeding of new crop varieties that are able to provide consistent yields even under salinity stress.

As a physiological response to osmotic challenges, plants close their stomata to reduce transpiration. As a result, the access of carbon dioxide is limited, while oxygen from water splitting accumulates in the mesophyll, leading to excessive photorespiration^7^. Millets (in *sensu lato*, comprising the tribus Paniceae and the subfamily of Panicoideae), as C_4_ plants, harbour great potential as climate resilient cereal crops, which meanwhile has also been recognised by policymakers. For instance, the Indian government has launched a nationwide campaign to promote millet production and consumption and declared 2023 the International Year of Millets (Government of India, 2023). From an economic point of view, the Sugar Millets (Panicoideae) play by far the dominating role compared to the millets in *sensu stricto* – the C_4_ plants sugarcane, maize, and sorghum range among the leading cereal crops. In contrast to maize and sugarcane, sorghum (*Sorghum bicolor* L.) can cope with water scarcity and, therefore, is suited for cultivation on marginal lands. Meanwhile, this resilient crop has moved into the top five cereals in terms of economic significance, which is linked with its versatile use for food, feed, and biofuel production^8^. Different types of *Sorghum bicolor* L. have been developed for different uses. For instance, grain sorghum stores abundant starch in its grains and is mainly used for food and feed, while sweet sorghum stores sugars in its stems and is suited for bio-ethanol production^9,10^. Thus, differences in source-sink relations determine different economic use.

Differential partitioning of sugars is not only the physiological trait defining the use of a given sorghum variety, but also a central mechanism to cope with osmotic stress as to maintain cellular turgor and to avoid wilting^8^. In most plant species, Sucrose is the dominant form of carbon transport and the most prevalent sugar in the phloem lumen^11^. The loading of sucrose into the phloem in the leaves occurs through the symplastically coupled sieve-element companion cell (SE-CC) complex. The route through the SE-CC is also used for unloading in the different sink tissues, including the growing vegetative tissues, such as roots and young leaves, and the stem storage sink, as well as the reproductive organs such as seeds^12^. The transition from the bundle-sheath cells to the SE-CC is apoplastic, though, which means that sucrose needs to leave the bundle-sheath cell by a membrane passage and enter the SE-CC by a second membrane passage. These membrane passages are achieved by virtue of sucrose transporters that actively load high quantities of sucrose into the phloem, such that the resulting concentration gradient will ensure bulk flow into the sink tissue^13,14^.

Since sorghum utilises apoplasmic phloem loading, sucrose transporters are of interest. SWEETs (Sugar Will Eventually be Exported Transporter proteins) are uniporters that transfer sugars along a concentration gradient. Members of SWEET clade III are crucial for the membrane passage from the assimilating cells into the phloem apoplast^15,16^. This transition is followed by entry into the SE-CC, via active transport since it has to work against the concentration gradient. This task is met by the Sucrose Transporters (SUTs), that co-transport protons exploiting the pH gradient between cell wall and cytoplasm that is sustained by the proton ATPases in the membrane^17^. The long-distance transport in the phloem is driven by the hydrostatic pressure gradient produced by the concentration gradient between source and sink.

Based on these mechanisms, the partitioning of assimilates between source and sink depends on several parameters^18^: (i) The rate of carbon fixation defining the activity of the source, (ii) the partitioning of triose-phosphates from carbon fixation between the formation of transitory starch in the plastid itself, and the export of sugars such as maltose or glucose from the chloroplast^19,20^, (iii) the transient storage of sucrose in the vacuole, and (iv) the import strength of the sink relative to the import activity of concurrent sinks. Under osmotic challenge conditions, sugars can either be used for osmotic adjustment in the source tissue itself^21^, in addition, under ionic stress they can be translocated to the roots and provide the energy required to sequester sodium ions into the vacuoles^22^.

As to be expected, the complex network of sugar sensing and transport is subject to hormonal regulation, with a prominent role for abscisic acid (ABA). For instance, the expression of sucrose transporters, and, thus, the partitioning of sugars, in response to different abiotic stresses is regulated by ABA-responsive transcription factors^23^. For example, the accumulation of sucrose in the leaves and roots of rice in response to drought and salinity is regulated by OsSWEET13 and OsSWEET15 as concluded from promoter-reporter studies^24^. Transactivation analysis showed that the two promoters are activated by the ABA-responsive transcription factor OsbZIP72^24^. Likewise, the sucrose transporters SUC2 and SUC4 mediate stress tolerance in *Arabidopsis thaliana* dependent on their activation by ABA^25^.

A previous study compared two sorghum varieties contrasting with respect to salinity tolerance^22^. The sweet sorghum variety Della was salt-tolerant, while the grain sorghum variety Razinieh was susceptible. This comparative study led to a working model, where the partitioning of sugars into the root of Della sustained a more efficient sequestration of sodium in the vacuoles of the distal elongation zone, such that the migration of salt into the shoot was slowed down. The resulting time gain allows the leaf to prepare metabolic adjustments that can mitigate the prospective challenge by excessive sodium. This model implies that source-sink partitioning of sugars might be crucial for salinity tolerance in sorghum. If so, one would expect that dynamic remodelling of sugar transport during development should be crucial for the response to salt stress. The most dramatic remodelling takes place during generative development, when inflorescence and seeds establish a new and strong sink competing with the roots for allocation of assimilates. Therefore, in the current study, we mapped spatial and temporal patterns of sugar partitioning and the expression of sugar transporters during vegetative versus generative development, and the response of these patterns to salt stress. From this map, we could identify two transporters as crucial factors. Then, using a dual-luciferase reporter assay in sorghum protoplasts, we can show that the promoter of *SbSWEET13*, responsible for export into the SE-CC apoplast, is induced by ABA, while the promoter of *SbSUT6*, driving the import into the phloem, is induced by MeJA. These patterns corroborate and extend models about systemic signalling inferred from our previous study^22^.

## Results

### The salt response of source-sink allocation is similar, but ground states are different

**Fig. 1:**
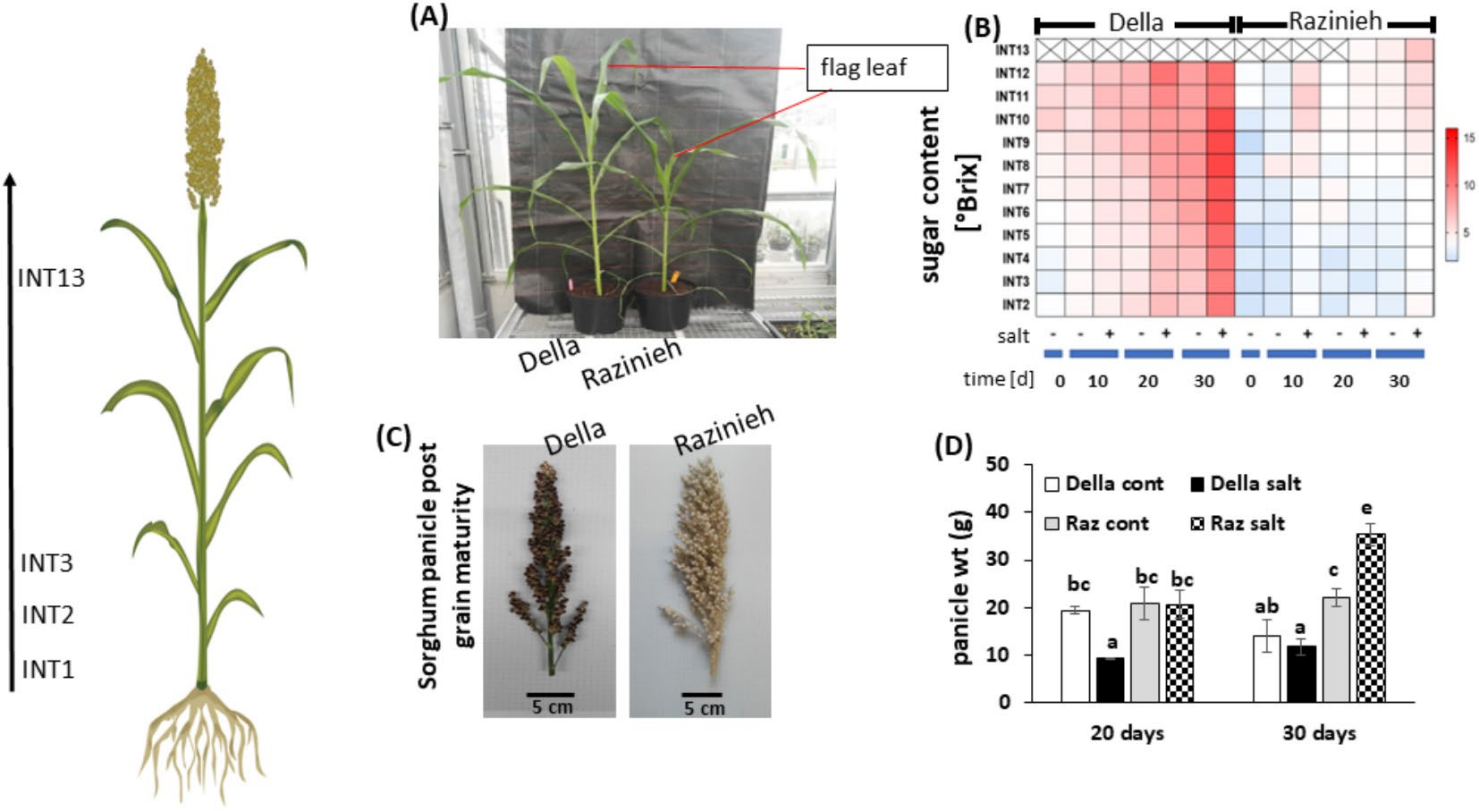
Effect of salt stress on phenotypic traits in two Sorghum genotypes contrasting in salt tolerance. Della is salt tolerant, Razinieh (Raz) is salt susceptible. (**A**) Morphology of sorghum plants at flag leaf stage grown under glasshouse conditions (the start of salt treatment, 0 day). (**B**) Content of soluble sugars (given as °Brix) in Della versus Razinieh at different time intervals after the onset of salt stress along the different internodes. (**C**) Appearance of the mature panicles after ≈ 2 months of salt stress. (**D**) Panicle weight at 20 days or 30 days of salt stress as compared to control conditions. Data represents mean and SE from 3 individual plants.

The genotypes Della and Razinieh had been shown to contrast with respect to salt tolerance during their seedling stage^22^. To test whether this difference is also present during maturation, we raised the plants under normal conditions, but commenced the salt treatment at the time of flag leaf emergence (Fig. 1A), such that the effect of salinity on maturation could be assessed. When we measured the content of soluble sugars separately for the different internodes (Fig. 1B), we observed a strong difference between the two genotypes, both with respect to the normal developmental progression as well as with respect to the salinity response. In Della, sugar content increased strongly from the time point of flag-leaf emergence, first in the apical internodes and subsequently moving towards the basal internodes. This increase was even further promoted under salinity, especially in the central internodes, and became prominent from day 20 after the onset of salt stress. In Razinieh, this pattern was seen as well, just at a much lower amplitude, both with respect to the gradient from apical towards basal internodes, as well as the stimulation of sugar content. Interestingly, this difference in sugar accumulation was not reflected in panicle development. In fact, the panicles of salt-stressed Razinieh appeared even fuller than those of Della (Fig. 1C), and this observation was confirmed when panicle weight was determined (Fig. 1D). While salinity caused a mild suppression of panicle weight in Della, it boosted panicle weight in Razinieh, by more than 50% compared to the control.

When we determined volume (Supplemental Figure S4A) and weight (Supplemental Figure S4B) of extracted juice as well as fresh weight for each internode individually (Supplemental Figure S4C), we found generally much higher values in Della. However, in contrast to sugar content, there was now a gradient with increasing values towards the more basal, older internodes. Moreover, juice volume was decreased under salinity. In other words: while sugar is concentrated in the younger, apical internodes, the older, basal internodes accumulate more water. Furthermore, under salinity, the juice is richer in sugar, but reduced in volume. These differences are more pronounced in Della, while Razinieh shows these responses at a strongly dampened amplitude.

When we recorded a developmental profile of the source parameters, leaf weight and area (Supplemental Figure S5A, C), we observed similar patterns with mildly elevated values in Della over Razinieh. Some differences were noted, however. For instance, Razinieh promoted growth in the uppermost two internodes at day 10 of salt stress and later even produced an additional internode, a phenomenon not seen in Della. On the other hand, leaf growth was promoted in the central internodes of Della under salinity from day 10 of salt stress. This increase was seen in Razinieh only from day 20 and at a lower amplitude. A corresponding profile of the sink parameters, internode length and diameter (Supplemental Figure S5B, D), showed a conspicuous elongation of the youngest two internodes in Della at the end of the experiment, but this boosted growth was seen in both, control, and salt-treated plants and, thus, simply represents a developmental response linked with flowering. Instead, Razinieh formed an additional internode at the end of the experiment and this final internode elongated significantly more under salinity. In contrast to internode length, we did not see significant differences in the pattern of internode diameter. Generally, in both genotypes, the basal internodes were thicker, as expected. Internodes in the apical half of the shoot became moderately thicker under salt stress with a tendency to a more pronounced response in Razinieh (that otherwise was generally slenderer as compared to Della).

Overall, both genotypes respond to salt stress by concentrating sugar in the younger internodes, while the older internodes incorporate water. The amplitude of this response is more prominent in Della and initiates from a significantly higher ground level as compared to Razinieh.

### Della can exclude sodium more efficiently from roots, mid internodes, and grains

**Fig. 2:**
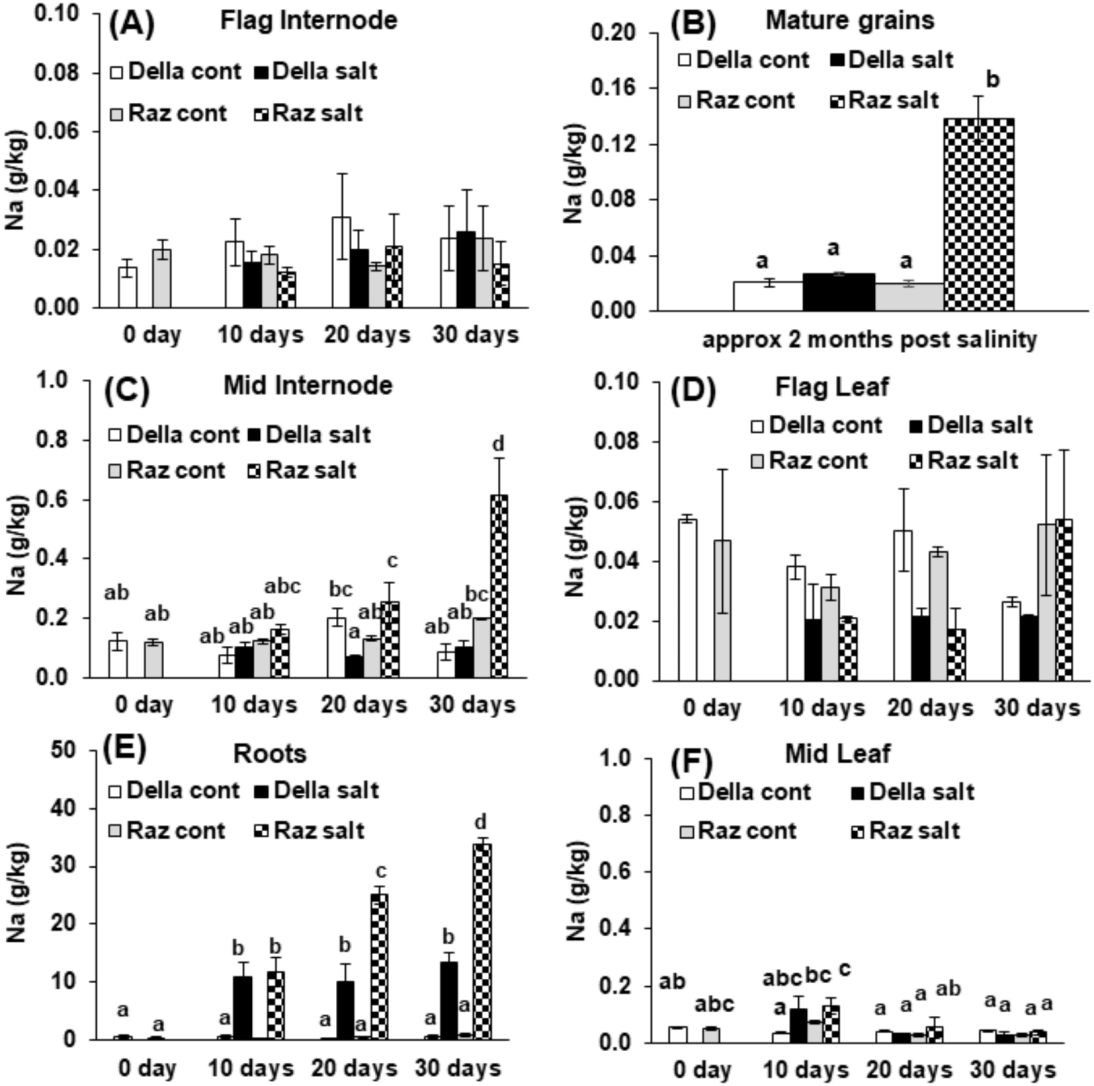
Sodium ion concentration in two Sorghum genotypes contrasting in salt tolerance. Della is salt tolerant, Razinieh (Raz) is salt susceptible. Steady-state levels of sodium ions are represented in (**A**) flag internode, (**B**) mature grains, (**C**) middle internode, (**D**) flag leaf, (**E**) roots, and (**F**) middle leaf in Della versus Razinieh at different time intervals after the onset of salt stress under control and salt treatment (100mM NaCl). Values represent the mean of at least three independent biological replicates ± SE. Different letters show significant differences between different genotypes and treatments according to Duncan’s test (*P <* 0.05).

To relate the observed differences in sugar partitioning (Fig. 1B) to the local challenge by salt, we followed the uptake of sodium and its allocation to different parts of the plant (Fig. 2). Both genotypes displayed a strong increase of sodium in the root, when assessed at day 10 (Fig. 2E), but progressively diverged when the exposure became longer. While sodium concentration increased steadily in Razinieh by around threefold of the value at day 10, it remained at this value of day 10 in Della. The sodium arriving from the root was mostly excluded from the mid internode, but this exclusion collapsed in Razinieh from day 20, while it persisted in Della (Fig. 2C). Very little sodium transported into the mid leaf, irrespective of the genotype (Fig. 2F). Likewise, in both genotypes, sodium was efficiently excluded from flag internode (Fig. 2A) and flag leaf (Fig. 2D). However, significant concentrations of sodium accumulated in the grains of Razinieh, but not in those of Della (Fig. 2B). Thus, there is a pronounced gradient of sodium from the root (high), over the mid internodes (intermediate), till the inflorescence (low) in both genotypes. The amplitude of this gradient is much higher in Razinieh as compared to Della.

Since accumulation of sodium is often accompanied by a reduction of potassium, all samples were also measured for their potassium content (Supplemental Figure S6). The highest concentrations of potassium were found in the central internodes (Supplemental Figure S6C) – in both genotypes it decreased over time, possibly due to uptake of water into the growing internodes. Resting levels were consistently lower in Razinieh by around 20%. Only at the end, at day 30, potassium decreased in Della to the same level. Under salt stress, the potassium levels decreased slightly in both genotypes, to about the values found for the resting level in Razinieh. The adjacent leaves (Supplemental Figure S6F) did not show salient differences between the genotypes except for the later stages of salt stress, when potassium became slightly elevated in Della, while being significantly (by around 30%) decreased in Razinieh. For the root (Supplemental Figure S6E), the pattern was inversed. Here, the resting levels were strongly increasing from day 10 to the experiment in Razinieh, but not in Della. As a result, potassium content at day 30 was almost 4-fold higher in Razinieh. This surplus was completely eliminated in response to salinity, while in Della, the (much lower) potassium level was maintained under salt stress. A similar pattern, albeit at lower amplitude, was seen for the flag internode (Supplemental Figure S6A) and the flag leaf (Supplemental Figure S6D). Here, the resting levels were higher in Razinieh, but decreased under salinity to the lower (and more or less constant) potassium levels in Della. Potassium levels in the grains (Supplemental Figure S6B) were more than an order of magnitude lower than in all other tested tissues and irresponsive to salt stress in Della. In Razinieh, however, the potassium level (comparable to Della in the control) doubled under salinity.

Overall, with exception of the root, potassium levels appeared well buffered against salinity in both genotypes. To get insight into potential mechanisms underlying the salient ion responses in the root, we measured the expression of ion channels responsible for the extrusion of sodium (*SbSOS1*), the sequestration of sodium into the vacuole (*SbNHX2*), and the retrieval of potassium (*SbHKT1*) in a time-course experiment (Supplementary Figure S7C). The ground levels of *SbSOS1* were doubled in Razinieh over Della but decreased rapidly in both genotypes to comparable residual levels independent of salinity. Likewise, *SbNHX2* and *SbHKT1* did not show any specific impact of salt stress, although ground levels of *SbHKT1* were elevated in Razinieh compared to Della. Overall, transcript levels of genes involved in sodium homeostasis in the root did not correlate with the observed patterns of ion partitioning.

### Salinity increases sink strength in Della, but not in Razinieh

**Fig. 3:**
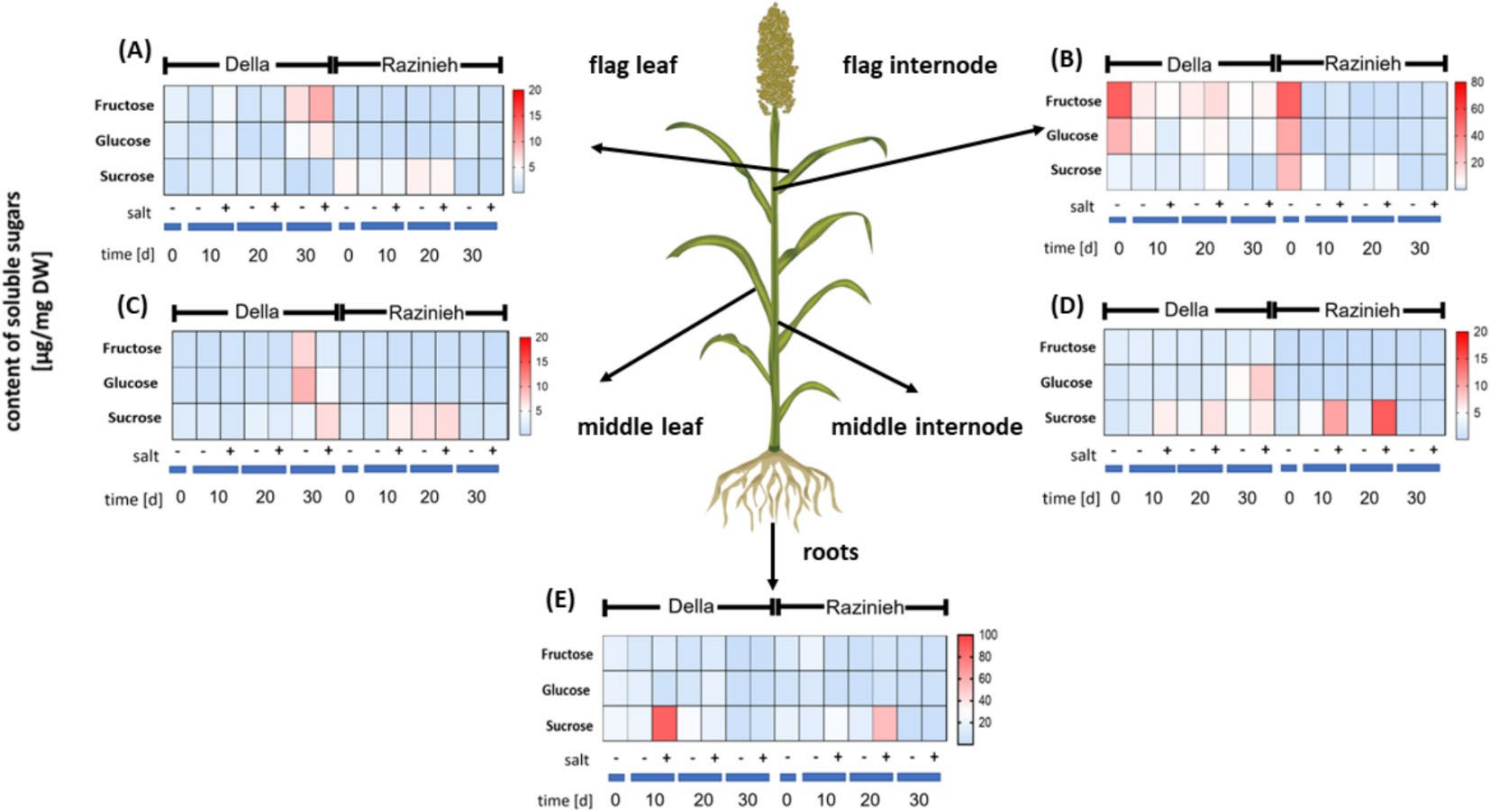
Differential partitioning of sugars under salinity in two Sorghum genotypes contrasting in salt tolerance. Della is salt tolerant, Razinieh (Raz) is salt susceptible. Steady-state levels of fructose, glucose, and sucrose in (**A**) flag leaf, (**B**) flag internode, (**C**) central leaf, (**D**) central internode, and (**E**) roots in Della versus Razinieh at different time intervals after the onset of salt stress. Data represents mean values from three biological replicates.

In the next step we probed source-sink relations under salinity for the transport of sucrose and its constituents, glucose, and fructose (Fig. 3). The root system is an important sink during salinity because sequestering and exclusion of sodium are energy dependent. In fact, a strong, but transient increase of sucrose was observed in both genotypes (Fig. 3E). However, this sugar import from the aerial parts was swifter and stronger for Della, as compared to Razinieh. Sugar concentrations in the generative sinks, flag internode (Fig. 3B) and flag leaf (Fig. 3A) showed a developmental dynamic with high initial levels of fructose and glucose in the internode that decreased strongly during further development. This decrease was not accompanied by a corresponding increase in sucrose levels. Thus, sucrose synthesis is unlikely to be responsible for this decrease. On the background of this developmental pattern, Della displayed one salient difference. Here, fructose accumulated in the flag internode at day 20 of salt stress, which was followed by a strong increase of fructose levels in the attached flag leaf at day 30 of salt stress. This phenomenon was not seen in Razinieh. The increase in fructose levels was accompanied with a significant, but weaker increase in glucose levels, while steady-state levels of sucrose did not rise. This indicates a swift activity of invertase and subsequent metabolisation of the sucrose imported into the generative sinks.

To relate these responses of sink tissues to the source, we analysed sugars in the internode (Fig. 3D) and the attached leaf (Fig. 3C). Here, a transient increase of sucrose in the internode of Razinieh under salinity was the most important difference. While such an increase was also seen in Della, the amplitude was much lower. Also, in the feeding leaf of Razinieh such an increase of sucrose was noted, albeit to a much lower extent as compared to the internode. In Della, a salt-induced increase of sugars was seen only at day 30.

Overall, Razinieh responds to salinity by accumulation of sucrose in source tissues, while this increase is not accompanied by a respective increase of sucrose in sink tissues. For Della, sucrose accumulation in the source tissue is less pronounced. Instead, sink tissues accumulate more sugars, roots in form of sucrose, generative sinks in form of fructose indicative of a more efficient partitioning of assimilates. In this context, it is interesting to look at the sugar content of mature grains (Supplemental Figure S8B). Here, sucrose was the dominant sugar, in Della accumulating to more than 60 mg.g^− 1^ dw, irrespective of salinity, while in Razinieh, sucrose under control conditions was only 38.7 mg.g^− 1^ but increasing strongly, to the levels found in Della, when plants were exposed to salinity.

From the other soluble sugars, only galactose showed a prominent change in response to salinity, particularly in the generative sinks (flag internode and flag leaf) after 30 days of treatment. Again, this increase was amplified in Della over Razinieh (Supplemental Figures S8A, D). A second prominent response was a developmentally induced, late, increase of galactose in source leaves (Supplemental Figures S8F) and the attached internode (Supplemental Figures S8C), as well as in the root (Supplemental Figures S8E). This increase was observed only for Della, but only in case of the root, this increase was promoted by salinity.

Overall, sink strength in Della is increased under salinity, which is not observed in Razinieh. This increase in sink strength is accompanied by concomitant increases in the level of galactose in the sink tissues.

### Sucrose transporters are more responsive to salinity in sorghum sink tissues

**Fig. 4:**
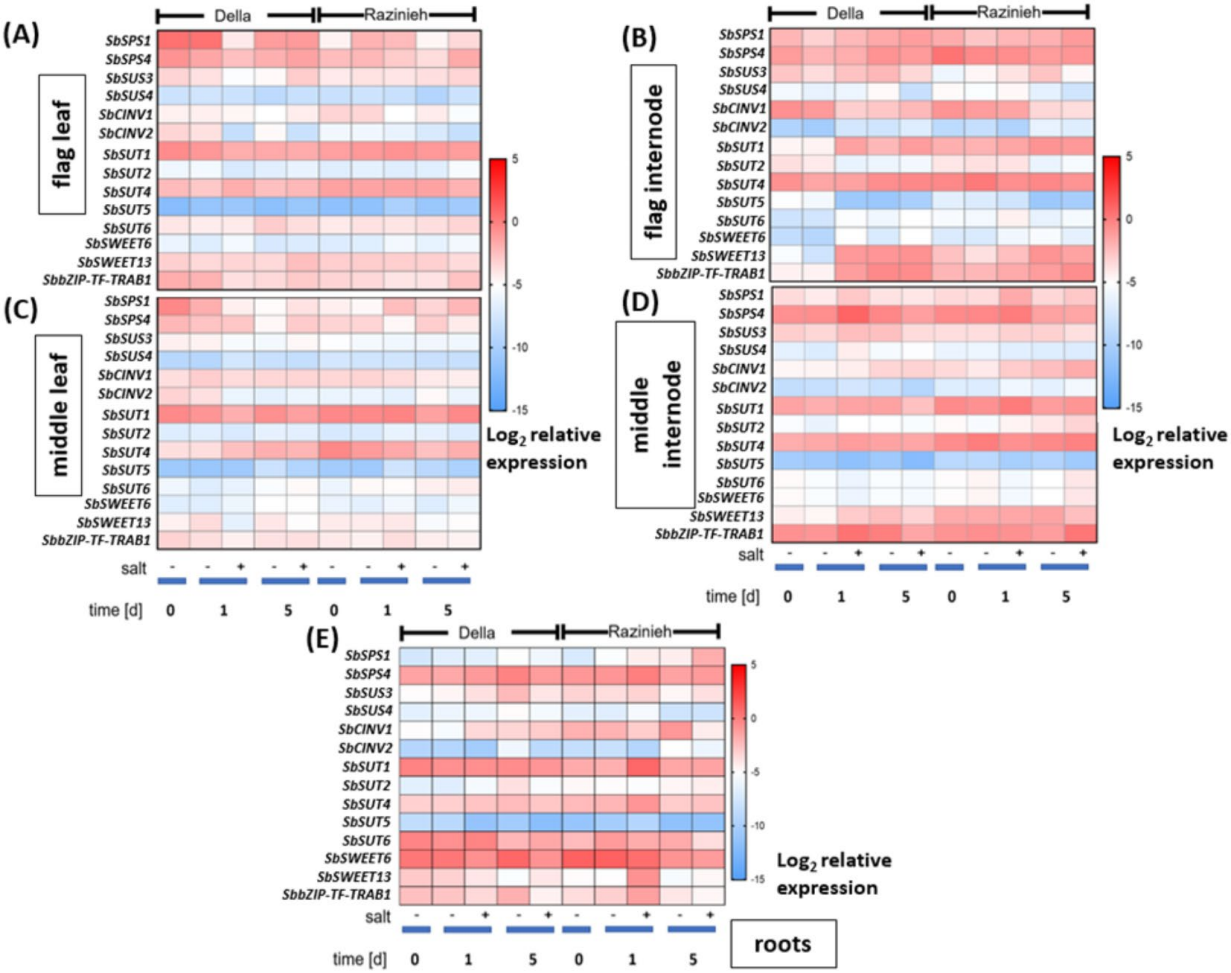
Differential expression of genes involved in sucrose metabolism and transport under salinity in two Sorghum genotypes contrasting in salt tolerance. Della is salt tolerant, Razinieh (Raz) is salt susceptible. Steady-state transcript levels in (**A**) flag leaf, (**B**) flag internode, (**C**) middle leaf, (**D**) middle internode, and (**E**) roots in Della versus Razinieh at different time intervals after the onset of salt stress are indicated as relative values based on the –ΔC_t_ method. Data represents mean values of three biological replications, each in technical triplicates, using ubiquitin and glyceraldehyde-6-phosphate dehydrogenase as internal standards. (SPS; Sucrose Phosphate Synthase, SUS; Sucrose Synthase, CINV; Cytosolic Invertase, SUT; Sucrose Transporter, SWEET; Sucrose Will Eventually be Exported Transporter, bZIP-TF-TRAB; basic leucine zipper transcription factor).

The partitioning of sugars depends on local metabolism in the source, active long-distance transport in the phloem mediated by sucrose transporters, and local metabolism in the sink. To find out, how the genes involved in these processes were regulated depending on genotype, tissue, and stress, we recorded temporal patterns of those genes. To priorities the plethora of genes possibly involved in source-sink partitioning, we made use of publicly available expression profiles. While suitable salinity-related profiles were not available, we could make use of drought-induced transcriptome responses of sorghum^24^. Here, a drought sensitive (IS20351) and a drought tolerant (IS22330) genotype were followed under the same conditions to test our hypothesis (NCBI GenBank: SRP073986), which allowed us to identify sucrose-related genes that were differentially expressed upon stress. After quality check, around 22.44 to 23.96 million high-quality clean reads per replicate could be obtained from the 12 libraries, and subsequently mapped against the sorghum transcriptome with a mapping rate of 85–88% (Supplementary Figure S9 and S10; Table S2). By Principal Component Analysis (PCA) a component linked with genotype could explain 64% of the variance (Supplementary Figure S11), while a second component accounting for 25% of the variance was mainly representing the impact of drought stress (Supplementary Figure S11). The impact of this second component was somewhat impaired by an outlier in the drought tolerant genotype. Nevertheless, it was possible to identify a large number of statistically significant gene candidates, as well as more than 6000 non-redundant genes (Supplementary Figure S12; Supplementary Table S7). Based on annotation and abundance changes (Supplementary Table S7), a set of promising candidates could be prioritised (Supplementary Table S8) for expression profiling by qPCR (Fig. 4). These included transporters, such as *SbSUT1*, *SbSUT2*, *SbSUT5*, and *SbSUT6*, metabolic enzymes, such as the sucrose synthase *SbSUS3*, as well as the sucrose phosphate synthases *SbSPS1* and *SbSPS4*, the cytosolic invertase *SbCINV1* and *SbCINV2*, but also a transcriptional regulator, binding to ABA-response elements (*SbbZIP-TF-TRAB1*). Interestingly, this transcriptional regulator had also been identified during a study on salt-responsive genes in rice^23^. These candidates were complemented by *SbSUT4* and *SbSUS4*, as remaining members of the respective gene families, and two members of the SWEET family known to be either salt-responsive in rice (SWEET13^23^), or upregulated in sweet versus grain sorghum (SWEET6^26^) .

The expression profiles obtained for these genes are, as to be expected, complex (Fig. 4), and depend on the type of gene, the stress, the tissue, and the genotype. To break this complexity down, we will follow the path of sugars from the source to the sink.

Sucrose phosphate synthases are the rate-limiting enzymes for sucrose formation in the source. Congruent with this function, *SbSPS1* shows higher expression in middle leaves (Fig. 4C) and flag leaves (Fig. 4A) of Della over Razinieh. Under salinity, this expression is rapidly, but transiently suppressed in Della in both leaves, while in Razinieh there is even a rapid, but transient induction in middle leaves. Instead, *SbSPS4*, which is less responsive in the beginning, initiates a late increase under salinity in flag leaves of Razinieh. Sucrose synthases provide the substrate for the biosynthesis of starch and cellulose. Here, expression of *SbSUS4* was generally low and not responsive, for *SbSUS3* despite the expression was low during developmental progression it exhibited a late responsive increase under salinity in Della. Cytosolic invertases break down sucrose into glucose and fructose and, thus, reduces part of the assimilates destined for long-distance transport for local consumption. Here *SbCINV2* was strongly but transiently downregulated under salt in flag leaves of Della, while, in Razinieh, this response was seen for *SbCINV1*. With dampened amplitude, this pattern was also observed in middle leaves.

For the respective internodes, *SbSPS4* is generally more active as compared to the leaves, irrespective of genotype and stress condition. Especially in the middle internode, there is a transient induction after one day of salt stress, most pronounced in Della, but also visible in Razinieh (Fig. 4D). *SbSPS1* showed a similar pattern here, although with a flattened amplitude. For the flag internode, both genes are strongly expressed. Compared with the flag leaf, modulations in response to salt were not pronounced (Fig. 4B). As for leaves, expression of *SbSUS4* was low in both, the middle, and the flag internodes, *SbSUS3* followed, again at lower amplitude, the pattern seen for *SbSPS1*. There was a slight induction later in development in both middle and flag internodes, which was suppressed under salinity, especially in Razinieh. In middle internodes, expression of *SbCINV1* was induced during later stages of salt stress, especially in Razinieh, while in flag internodes, expression was high during early time points in both genotypes, generally dropping subsequently. The second invertase, *SbCINV2* was low expressed, not reaching the levels seen in the adjacent leaves.

For the root, *SbSPS4* was the dominating sucrose synthetising transcript, but was expressed more or less constitutively (Fig. 4E). *SbSPS1* instead became relevant only in the final stage of salt stress in Razinieh. For the two sucrose synthases, *SbSUS3* was relevant and showed a slight induction under salinity in Razinieh, but not in Della. Among the invertases, transcripts for *SbCINV1* transcripts were dominating. Here, expression in Razinieh was generally more elevated, with a downmodulation under salinity.

A comparison of the expression patterns for genes involved in sugar metabolism (Fig. 4) with the actual distribution of sugars (Fig. 3) did not reveal obvious correlations. Instead, there are salient points with respect to the expression of sugar transporters. Here, the high ground levels of *SbSUT6* and *SbSWEET6* in the root are noteworthy (Fig. 4E), because both genes show only low expression in all other tissues. The strong early accumulation of sucrose in roots of Della, not seen in Razinieh (Fig. 3E) correlates with a difference in the resting level of *SbSUT6*, which is higher in Della as compared to Razinieh (Fig. 4E). Meanwhile, in flag internodes, *SbSWEET13* and the ABA-responsive transcription factor *SbbZIP-TF-TRAB1* are more responsive to treatment in Della compared to Razinieh (Fig. 4B). Conversely, *SbSUT1* and *SbSUT6* are generally elevated in flag internodes under treatment, more rapidly in Razinieh as compared to Della (Fig. 4B). In parallel, *SbSUT2* is downregulated in Della early during salinity, which is not seen in Razinieh. Since grain filling requires passage of carbohydrates through the flag internode, the expression of these three genes can be used as proxy for grain filling. The expression patterns for these genes correlated with the strong increase in panicle weight under salinity in Razinieh (Fig. 1D).

Thus, our data do not show a scenario, where genes involved in sugar synthesis are induced in source tissues. Instead, we find a response of sucrose transporters in sink tissues. Here, the early expression of *SbSUT2*, *SbSUT6*, and *SbSWEET13* heralds the subsequent path of the sugar. This led to the question, whether differences in the promoters of these three genes might explain the observed differences in assimilate partitioning and salinity responses of the two sorghum genotypes.

### ***Cis***-element comparison predicts altered jasmonate responses for the ***pSWEET13*** promoter

**Fig. 5:**
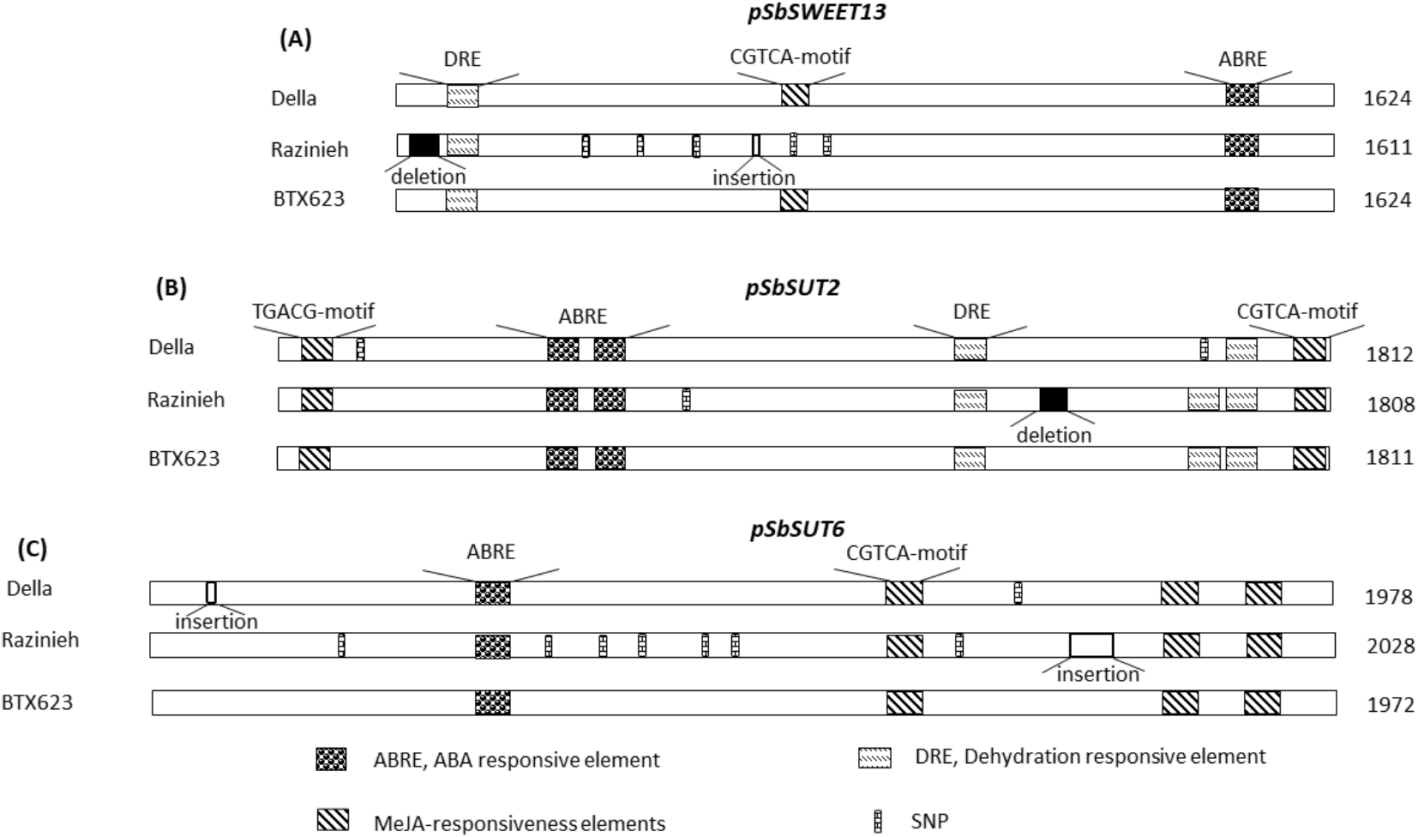
Survey over relevant cis-acting motifs predicted for the promoter sequences of the sugar transporters *SbSWEET13*, and *SbSUT2* and *6*, from two Sorghum genotypes contrasting in salt tolerance (Della: salt tolerant, sweet type, Razinieh: salt susceptible, grain type) along with the genotype BTX623 (grain type, genotype for the *Sorghum* reference genome). In addition to putative motifs for the response to ABA, MeJA, and drought, Single Nucleotide Polymorphisms (SNPs) informative for Razinieh, are indicated analysed with PlantCARE algorithm.

To get insight into the regulatory features of three candidate sucrose transporter genes, we cloned all six promoter regions (*pSWEET13*, *pSUT2*, and *pSUT6* from both Della and Razinieh) 1600 ~ 2000 bp upstream of ATG start codon. Sequence analysis predicted several *cis-*acting elements for abiotic stress responses (Fig. 5). These included the motif ACGTG known as abscisic acid responsive element (ABRE), the methyl-jasmonate responsive elements CGTCA and TGACG, dehydration responsive elements (CCGAC), and ACGTG, which is a putative binding site for bZIP transcription factors (Fig. 5). The incidence of these putative *cis-*elements differed between the three genes. For instance, the ABRE motif was found once in the *pSbSWEET13*, as well as in the *pSbSUT6* promoter, but was duplicated in the *pSbSUT2* promoter. Also, the dehydration response element CCGAC is abundant in *pSbSUT2*, but missing from *pSbSUT6* and only present in one copy in *pSbSWEET13*. Instead, *pSbSUT6* harboured three copies of the putative methyl-jasmonate response element CGTCA, while *pSbSUT2* had only one copy of this motif, complemented by TGACG, the alternative methyl-jasmonate responsive motif. The most salient, potentially functionally relevant, difference between the genotypes was a missing CGTCA motif in the *pSbSWEET13* allele of Razinieh, and an additional dehydration motif in the *pSbSUT2* Razinieh allele. The full alignment with the details of these *cis-*elements is given in Supplementary Table S9**.**

### ABA induces the ***SbSWEET13*** promoter, MeJA the***SbSUT6*** promoter

**Fig. 6:**
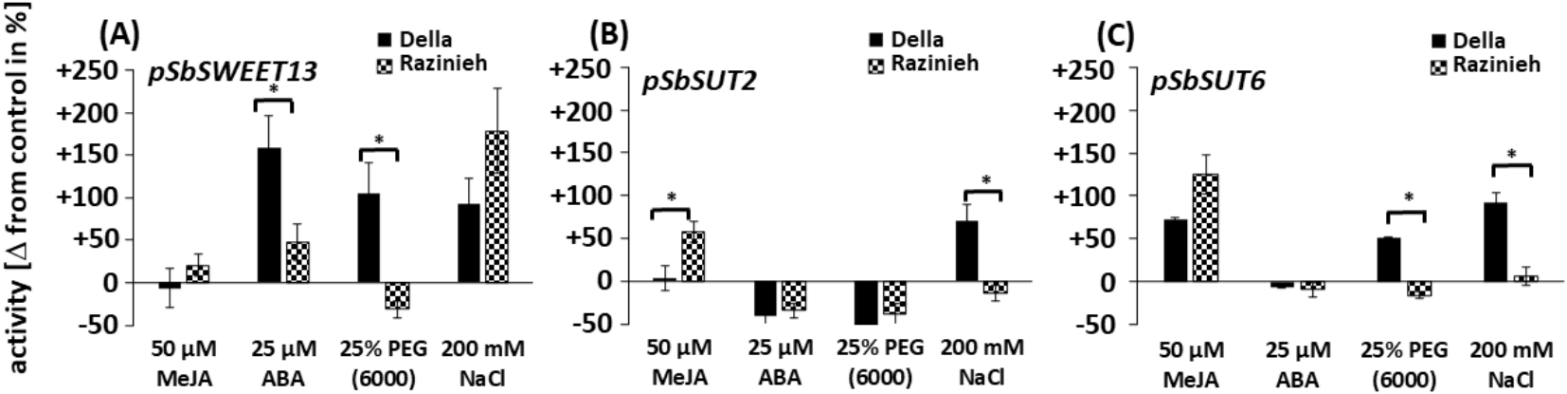
Response of promoter activities for three sugar transporters deriving from two Sorghum genotypes contrasting in salt tolerance. Della is salt tolerant, Razinieh is salt susceptible. Change of promoter activity measured 1 h after the onset of the respective treatment compared to the control condition in % of this resting activity. (**A**) Sucrose Will eventually be exported transporter 13 (SWEET13), (**B**) sucrose transporter 2 (SUT2), (**C**) sucrose transporter 6 (SUT6). Promoter activities were determined using a dual luciferase reporter system. Promoter activities were modulated by the hormones methyl jasmonate (MeJA), and abscisic Acid (ABA), by osmotic stress with polyethylene glycol 6000 (PEG 6000), and by salinity (NaCl). Data represents mean values of three independent experimental series. Significance between the responses of the two alleles were tested by a Student‘s t-test, * significant at *P* < 0.05.

To test, whether the differences with respect to the putative *cis-*acting elements in the promoters of the analysed sugar transporters are functionally relevant, we established a dual-luciferase promoter-reporter assay system in suspended sorghum protoplasts and measured the inducibility for the promoter alleles cloned from either Della or Razinieh for *SbSWEET13*, *SbSUT2*, and *SbSUT6* by either ABA (25 µM), MeJA (50 µM), osmotic stress 25% w/v PEG 6000 (-0.735 MPa), or salinity (200 mM NaCl) comparing to a mock treatment, which did not induce any significant activation in the activity of the promoters (Fig. 6**)**.

We observed a strong induction of the Della allele for *pSWEET13* by 150% in response to ABA (Fig. 6A), while the Razinieh allele was only induced by 50%. In contrast, MeJA was not effective for either of the two alleles. Osmotic stress induced the Della allele of this promoter by 100% but failed to induce the Razinieh allele. In contrast, salt stress was more inducive for the Razinieh allele, but significantly weaker for the Della allele. Thus, the absence of *bona-fide* jasmonate response elements in the Razinieh allele of *pSWEET13* was correlated with a reduced inducibility by ABA and osmotic stress, but an elevated inducibility by salinity.

For the *SbSUT2* promoter (Fig. 6B), we did not observe any induction by ABA or osmotic stress, although ABRE and drought-response elements were predicted to be more abundant in this promoter. Instead, there was a significant induction by MeJA (50%), but only for the Razinieh allele. In contrast, salinity induced only the Della allele (50%). Overall, although the *SbSUT2* promoter harboured a duplicated ABRE motif and several copies of the dehydration motif, it was neither induced by ABA, nor by osmotic stress. With respect to allelic differences, an additional copy of the dehydration motif in the Razinieh allele correlates with induction of MeJA (missing in the Della allele lacking this additional motif) and a missing response to salinity (seen in the Della allele).

Among the three promoters investigated, *SbSUT6* was showing the strongest induction by MeJA (Fig. 6C), especially for the Razinieh (by about 150%). This correlates well with the higher number of the putative methyl-jasmonate response element CGTCA found in this promoter. Instead, despite presence of a putative ABRE element, there was no ABA response whatsoever. The Della allele of this promoter was moderately induced by osmotic stress (by about 50%) and salinity (by about 100%), while the Razinieh allele was not responsive. This contrasts with an identical set of *cis*-elements but might be linked to a specific insertion not far upstream from the transcription start in the Razinieh allele and a different, smaller, insertion specific for the Della allele around 1800 bp upstream of the transcription start.

Overall, the predicted *cis*-elements were partially reflected by the regulation pattern of promoter activity (inducibility of *pSWEET13* by ABA and osmotic stress; inducibility of *SbSUT6* by MeJA). Allelic differences in *cis*-elements and differences in regulation point to a role for a putative jasmonate response motif, CGTCA, for the inducibility of *pSWEET13* by ABA and osmotic stress (linked with a reduced inducibility by salt). Likewise, a higher abundance of the same motif in *pSUT6* correlated with a higher inducibility by MeJA, matching the bonafide function of this *cis*-element. The responses between exogenous hormones and abiotic stress were mostly different, indicating that these stresses deploy alternative signals in addition to triggering a hormonal response.

## Discussion

**Fig. 7:**
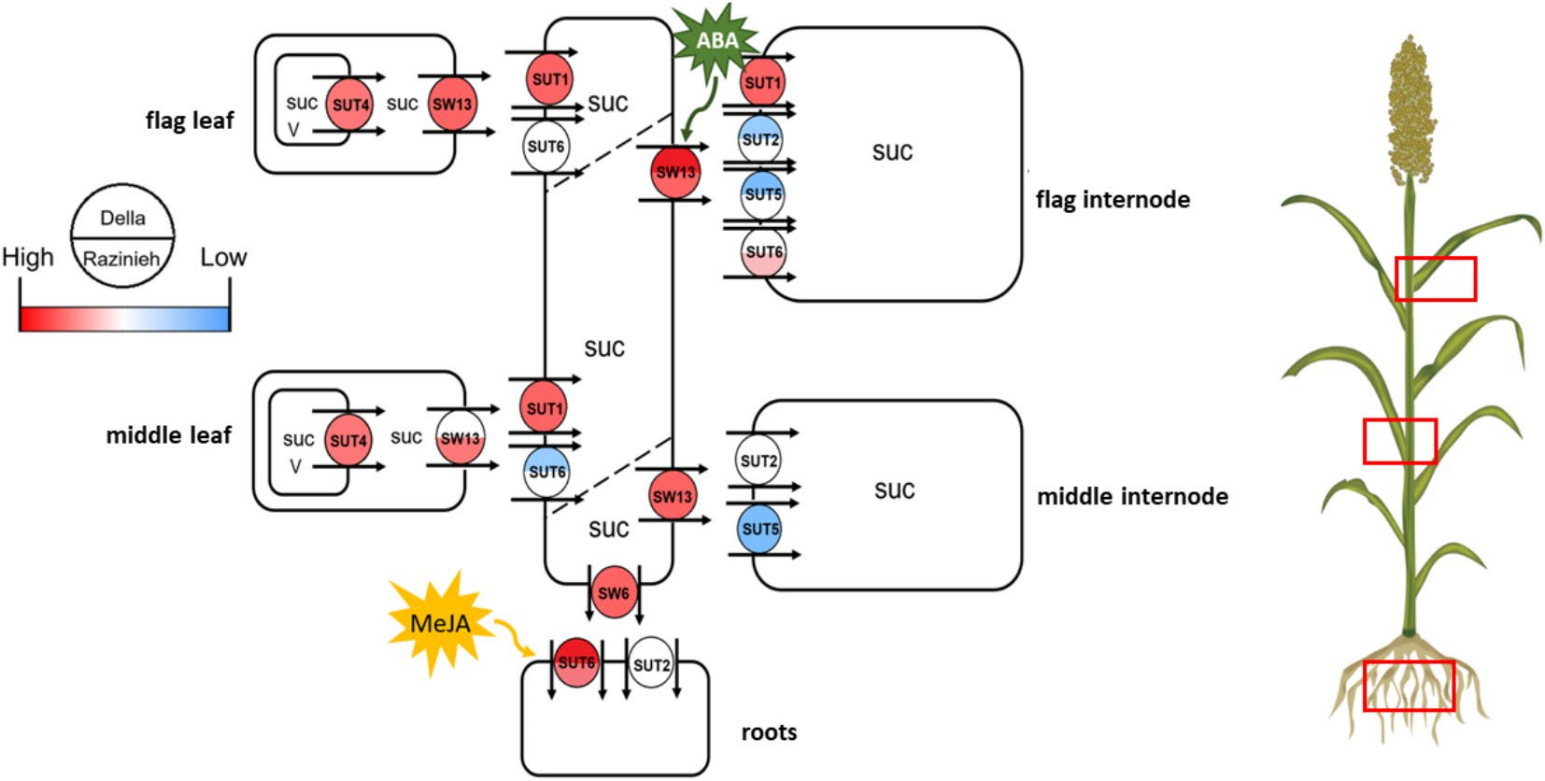
Working model describing the regulation of sucrose (SUC) partitioning under salinity in two sorghum genotypes contrasting in salt tolerance during grain filling. Della is salt tolerant, Razinieh (Raz) is salt susceptible. (SUT sucrose transporter, SW sucrose will eventually be exported transporter).

Our previous study on the same pair of genotypes^22^, had shown that, during vegetative development, a rapid translocation of sucrose into the root of Della correlated with a more efficient sequestering of sodium in the elongation zone, giving the shoot more time to prepare against the advent of sodium. This might be the reason why Della can cope better with salinity as compared to Razinieh, where these processes were either slower or less efficient. We have now extended this working model to the generative development, asking, how repartitioning of source-sink relations might contribute to salt tolerance. In fact, we see that the two genotypes respond differently to salinity in terms of resource allocation – Della partitioned sugar into the stem and roots, Razinieh into the grains instead. While these differential patterns did not match the expression of sugar metabolic genes, we found a differential expression in the sink tissues (Fig. 7). Here, unloading of sucrose in the root was linked with elevated ground levels of *SbSUT6* and *SbSWEET6* in Della, while *SbSWEET13* and the ABA-dependent transcription factor *SbbZIP-TF-TRAB1* correlated with sucrose unloading in the internodes in the same genotype. In contrast, expression of *SbSUT2* in flag internodes accompanied promoted panicle development in Razinieh. For *SbSWEET13* and *SbSUT6*, these patterns were linked with differential activation of the respective promoters ABA and MeJA as reported by a dual-luciferase assay in sorghum protoplasts. These data lead to the following questions that will structure the discussion:

- What is the functional context for the differential partitioning of sugars?
- What is the role of the root for the resource allocation in the shoot?
- What is the mechanism underlying sink expression of sugar transporters?

## Resource allocation under stress – the decision depends on the strategy

Under stress, plants need to render a strategic decision – they can use their resources to restore physiological homeostasis and, thus, to sustain the survival of the individual. Alternatively, they can redirect their resources to produce offspring in form of seeds that can, due to their reduced metabolism, survive even under adverse conditions to re-launch the next generation once the situation has returned to favourable conditions. Sorghum is a very interesting model in this regard since breeding has led to types differing in resource allocation. The original grain sorghum has been re-wired into sweet sorghum by repartitioning sugars from the grain into the internode sink. This transition is linked with recessive mutations in the *DRY* locus, leading to a deletion or loss-of-function of *Sb006G147400*, a transcription factor belonging to the *NAC* family^27^. This clade of NAC factors acts as master switches for cell wall synthesis^28^ and control a second tier of MYB transcription factors that, in turn, regulate genes involved in cell-wall synthesis, such as cellulose synthases, or genes integrating cell wall synthesis into development, such as xylem-specific protease executing programmed cell death during vascularisation^29^. Since cellulose is composed of sugar residues, it is not surprising that the state of the cell wall feeds back on the expression of genes involved in sugar metabolism, transport, and response^30^. This feedback plays an important role during fruit ripening, where the softening of the cell wall needs to be orchestrated with the accumulation of sugars, explaining the important role of NAC transcription factors for fruit quality^31^. This functional nexus between cell wall and sugar accumulation seems to be conserved in all Angiosperms and would explain, why the loss-of-function of *DRY* in sweet sorghum not only impairs the thickening of secondary walls in parenchymatic cells of the internode, but also causes the juicy appearance of the sweet sorghum pith. This will not only divert assimilates towards the internodes as a major sink, but also lead to a different response to salinity stress as compared to the ancestral grain sorghum. While Della accumulated sugars in the central internodes when it was confronted with salinity, Razinieh produced heavier panicles. Accumulation of sugars and other compatible solutes is a widespread adaptive response to salinity^32^. The absence of a *DRY* function in sweet sorghum might promote this response even further, because sugar accumulation is uncoupled from cellulose synthesis. This phenomenon is not limited to Della but can also be observed in other sweet sorghum genotypes^33^ and allows to sustain metabolic adjustments that safeguard photosynthetic activity, such as suppression of photorespiration, or buffering redox homeostasis^22^.

This leads to the question, of how the different salt response in Razinieh can be explained. Did this grain sorghum genotype acquire a novel signal pathway that is missing in Della? Although conceivable, there is a more parsimonious hypothesis. The pattern seen in Razinieh might simply be the default pathway – while perennial plants need to sustain vegetative growth under stress, annuals like sorghum have a more efficient alternative: promote generative development to produce seeds at any costs and, thus, avoid the fitness costs coming from individual stress adaptation. Grain filling in cereals and leaf senescence are interdependent. For instance, partial degraining of wheat can delay leaf senescence^34^. On the other hand, mobilisation of nitrogen during leaf senescence promotes grain filling^35^. This resource transfer to the grain is again controlled by a (different) NAC transcription factor^36^. The more pronounced salt-induced necrosis in Razinieh is, therefore, expected to promote the translocation of assimilates into the grain, while in Della, the internodal sink will intercept this translocation.

### Roots of resilience – what is the role of sugar transport?

A comparative study on salt and sugar allocation during vegetative development^22^ revealed sequestration of sodium into the vacuoles of the root elongation zone as crucial factor for the superior salt tolerance of Della over Razinieh. This correlated with a slower penetration of sodium into shoots and leaves, which allowed Della to deploy anticipative metabolic adjustments buffering photosynthesis against the perturbations from the arriving sodium.

The sequestration of sodium into the plant vacuole requires the activity of the sodium-proton antiporter^37^ and, thus, depends on the proton gradient across the tonoplast, which is sustained by proton ATPases^38^. The rapid accumulation of sucrose in salt-stressed Della (Fig. 3E) may, therefore, not only assist in maintaining turgor in the root, but also provide the energy needed for sodium sequestration.

Generally, the levels of photoassimilates including sucrose, fructose, and glucose in both source and sink tissues did not indicate a reduction in sugar production under salt stress; in some cases, these levels were even elevated (Fig. 3). This finding reduces the likelihood of impaired photosynthetic efficiency, aligning with studies suggesting that salt stress does not universally damage photosynthesis but may instead trigger specific acclimation responses, such as osmoprotectant synthesis and sugar accumulation^39,40^. This rather shifts the focus to the regulatory mechanisms governing sugar allocation and transport, particularly sucrose, which play a critical role in plant stress tolerance^22,40,41^.

Transcripts for a sugar transporter responsible for the salt-induced rapid accumulation of sucrose in Della roots, should meet two criteria: (i) Their ground level prior to salt stress should be higher in Della as compared to Razinieh. (ii) Their induction by salinity should be more pronounced in Razinieh as compared to Della because their insufficient ground level needs to be compensated under salinity. Among the probed transcripts, *SbSWEET13* most clearly meets these criteria and, therefore, must be seen as prime candidates to explain the sucrose accumulation in Della (Fig. 4E). Interestingly, the promoter of *SbSWEET13* is induced by ABA in Della, only to a much weaker extent in Razinieh (Fig. 6A). The transcriptional regulator *SbbZIP-TF-TRAB1*, binding to ABA-response elements, shows the same regulatory features as *SbSWEET13*, i.e., higher ground level in Della, stronger induction under salt stress in Razinieh. In contrast, among the *SUT* genes found to be expressed in the root, only *SbSUT1* fully meets the two criteria defined above, while *SbSUT6* does not display salt induction in Razinieh, and *SbSUT2*, due to a much lower abundance; seems irrelevant in this context.

The different regulation patterns reflect the different functions for these transporters^42^. While SUTs load sucrose into the companion cells of sieve elements exploiting the energy from a proton gradient across the membrane to transport their cargo against its concentration gradient, SWEETs facilitate passive transport following the concentration gradient. Considering these molecular mechanisms, one would expect SUTs to be expressed in source, SWEETS in sink tissues. In fact, for rice, with exception of weak expression of one member, transiently detected in seminal roots, the root does not show SUT expression^43^. However, under drought stress, cases of SUT expression have been observed for roots of *Arabidopsis thaliana*^44^ or maize^45^. This has been interpreted in the context of retrieval of sugar leaked into the apoplast, or vacuolar mobilisation of sugars in the root itself. However, one has to keep in mind that SUTs do not only transport sucrose, but also accept other cargoes, including various glycosides^46^. A further *caveat* is that the root has been systematically neglected as compared to the aerial parts of the plant.

Albeit a mismatch is observed between *SbSWEET13* expression and sugar content under salt stress at the flag internodes in Della (Fig. 3B), this observation aligns with the complex dynamics of eukaryotic regulatory systems. Post-transcriptional mechanisms and feedback regulation often decouple gene expression from functional output^47^. Such divergence likely represents a compensatory feedback loop, wherein reduced sugar content triggers increased *SbSWEET13* expression as part of a homeostatic mechanism to restore cellular balance.

Nevertheless, also the functional specificities of the transporter, shift *SbSWEET13* into the limelight with respect to the rapid accumulation of sucrose in the root of Della. Also, in seedlings of *indica* rice, the homologous *OsSWEET13* and *OsSWEET15* were strongly induced in the root within hours after the onset of salt stress^23^ corroborating a central role of these transporters for salinity tolerance. This stimulates the question, of by what mechanism can Della deploy a more efficient expression of *SbSWEET13?*

### Sugar and salt – jasmonates as a hidden player?

Allelic differences in *SbSWEET13* inducibility (Fig. 6A) and predicted *cis*-elements (Fig. 5A) connect induction by ABA and osmotic stress with the presence of a CGTCA motif in Della, while absence of this motif in Razinieh correlates with stronger salt inducibility. The regulation of *SbSWEET13 in planta* correlates with that of *SbbZIP-TF-TRAB1* (Fig. 4). The transcriptional activator TRAB1 was originally discovered in rice^48^ as missing link explaining the activity of VIVIPAROUS. While VIVIPAROUS by itself does not bind to ABA responsible elements, it can act indirectly by activating TRAB1, which in turn can deploy downstream genes through their ABA responsible *cis*-elements. In fact, all three tested promoters (*SbSUT2*, *SbSUT6*, and *SbSWEET13*) harbour such elements (Fig. 6), but these are identical for the two genotypes and, therefore, cannot account for the differential regulation. The only tangible allelic difference is the CGTCA motif. However, this motif is not linked with inducibility by ABA, but by MeJA^49^.

This runs across with the close parallelity in the regulatory patterns of *SbSWEET13* and *SbTRAB1* indicating a role of ABA. There are two scenarios to resolve this discrepancy:

1. The two hormones have been shown to interact during osmotic challenges in a couple of plant models. For instance, drought-induced accumulation of ABA in roots of *Arabidopsis thaliana* was eliminated in mutants, where jasmonate biosynthesis was disrupted^50^. On the other hand, ABA accumulation in response to osmotic challenge was only mildly reduced in a rice mutant, unable to accumulate any jasmonates due to a loss of function of allene oxide cyclase^51^, and the same was observed for the induction of ABA in response to salt tress^52^. On the other hand, ABA can modulate jasmonate synthesis, since the phospholipase A required for the release of α-linolenic acid in the plastid membrane as first substrate of oxylipin biosynthesis is induced by ABA^53^. Thus, induction of ABA as reported by TRAB1 might activate jasmonate biosynthesis, which, in turn, deploys *SbSWEET13* expression.
2. However, a simpler scenario would deduce the induction of *SbSWEET13* directly from the rapid and strong induction of jasmonate biosynthesis in response to salinity^52^. If so, the response of TRAB1 might be a parallel phenomenon or even a consequence of the jasmonate induction. In this context, it should be noted that jasmonates have been shown to induce the expression of the ABA receptor PYL4 in *Arabidopsis thaliana*^54^.

Since corresponding mutants are not available for Sorghum, an alternative strategy to discriminate between the two scenarios would be to follow the early time course of hormonal accumulation in the roots of Della and Razinieh.

### Outlook

As outlined earlier, salt tolerance in sorghum is associated with a delay of sodium translocation from root to shoot, allowing the shoot more time to prepare for the influx of sodium^22^ and here we report an unbalanced sugar distribution manifested by differential sucrose transporters expression at generative development. In fact sugar transporters activity serves as a key target for traditional or targeted breeding methods, aiming either to enhance phloem loading or to boost sink strength in crop species^55^. We seize this opportunity to emphasize the importance of conducting more field or natural habitat experiments to bridge the gap between analytical plant sciences and agronomy and breeding research, as there is often a lack of consistency between those fields^56^. Finally, we conclude that generation of plants or selection of genotypes with improved resource allocation under stress conditions, can lead to increased phloem sucrose shuttling to specific sink tissues according to the crop type. Perhaps generation of SUT6, SWEET13 or, (specific) NAC transcription factor overexpressor plants can increase our understanding of resource transfer to roots and internodes, or grains instead. Investigating monosaccharide SWEET transporters clade (II) and sucrose transporters in inflorescence tissues^14,57,58^, in addition to vacuolar invertases vacINVs and vacuolar transporters also would be needed to shed light on sugar subcellular concentration role in increasing sink strength under stress conditions.

## Methods

### Plant growth conditions

 The study compared two varieties of *Sorghum bicolor* (L.) Della (sweet sorghum), and Razinieh (grain sorghum). Sorghum plants were grown in the greenhouse at the Experimental Station of the Joseph Kölreuter Institute for Plant Sciences at of the Karlsruhe Institute of Technology (Karlsruhe, Germany) during the summers 2019 and 2020. The surface-sterilised caryopses were sown and raised in 5-L plastic pots filled with Floraton 3 (Floragard Vertriebs GmbH, www.floragard.de) soil for 6 weeks. Subsequently, seedlings were transferred into 10-L pots filled with peat-based substrate (Tonsubstrat; Klasmann-Deilmann; http://www.klasmann-deilmann.com) and remained until the end of the experiment in the glasshouse with temperatures sustained at 25/22 ± 3 °C during day/night, respectively, with a mean relative humidity of ≈ 20/40% during day/night, while maintaining a continuous flow of fresh air during the experiment. Plants were exposed to a 12-h photoperiod complementing ambient light to about 1000 µmol m^− 2^ s^− 1^ Photosynthetically Available Radiation by fluorescent bulbs (400 W / 220 E40 55,000 lm; SON-T AGRO, Philips) installed at 3 m above the pots. Seedlings were thinned to one per pot after one week of germination. Plants were irrigated to maintain 80% of field capacity throughout, from the first day of cultivation until the end of the experiment. Salinity stress was administered from the emergence of the flag leaf, which is the final and uppermost leaf that develops on the stem stalk by applying 400 mL of a 100-mM NaCl solution over a period of 2 months until plants reached grain maturity. Concurrently, a mock control was run, where the plants were treated in the same way by de-ionised water but omitting the salt.

### Phenotyping

 Morpho-physiological parameters were recorded from the start of salt treatment in intervals of 10 days. In addition to the number of internodes and leaves, the following parameters were determined individually for each internode: fresh weight (g), length (cm), and blade area of the adjacent leaf (cm^2^) were recorded. Then, the juice was extracted for each internode using a traditional cane crusher (VEVOR Juicer 110LBS/H, India) to determine juice yield as volume (mL/internode) and weight (g/internode), along with the concentration of sugar in the extracted juice (°Brix) using a manual refractometer (Model PAL, Atago Co. Ltd., Tokyo, Japan). Data represents mean and standard errors from three biological replicates.

### Determination of sugar accumulation and ion content.

 Samples were harvested from representative parts selected along the entire plant: roots, central internodes with adjacent middle leaves, and flag internodes with adjacent flag leaves at day 0 (untreated plants used to determine the ground level), then from both control and treated plants after 10, 20, and 30 days of salinity treatment. After excision, the sampled plant parts were washed gently several times with de-ionised water and divided into two groups to determine sugar composition and ion content, respectively.

### Determination of sugar content and composition

The washed samples were immediately frozen in liquid nitrogen and kept at − 80 °C till metabolite extraction according to^59^. Sugar content and composition were determined against the respective sugar standards by GC-MS (GC/MS/MS Agilent 7890 A / 5975 C / Chromtech Evolution 3, Agilent, Santa Clara, USA) as described in^22^.

### Measuring the content of sodium and potassium ions

The harvested samples were incubated at 80℃ in a drying oven for three days until they had reached a constant weight. Then the dry tissues were homogenised into a fine powder (TissueLyser, Qiagen) and aliquots of ~ 50 mg from each sample were digested in a heating block (~ 110 °C) in closed vessels by HNO_3_ (65% v/v, subboiled) and H_2_O_2_ (30% v/v, p.A.). The digest was diluted in 50 mL of ultrapure water. Subsequently, the digested sample was used for measuring the ion content by Inductively Coupled Plasma Optical Emission Spectrometry ICP-OES (715ES, Varian). For more details on digestions and measurements please refer to^22^.

### RNA extraction, cDNA synthesis and quantitative real-time PCR

Gene expression was measured in all parts of the plant, sampling after 0, 1 and 5 days of salt treatment. Tissue samples were excised from roots, the middle and flag internodes as well as from the adjacent leaves. The internodes and the leaves were quickly sectioned at their midpoint eliminating the mid-rib with a sharp blade. The sampled tissue was instantly frozen in liquid nitrogen and kept at -80 °C until processing. Total RNA was extracted and reversely transcribed into cDNA synthesis as described in^52^ using the transcripts for *Ubiquitin* (*SbUBQ*) and *Glyceraldehyde-6-phosphate dehydrogenase* (*SbGAPDH*) transcripts as internal standards. Relative transcript levels between the different samples were compared using the 2^−∆^C_t_ method^60^. Data represents the geometrical means from three biological replicates, each in technical triplicates. The details of the oligonucleotide primers to amplify the genes of interest are shown in Supplementary Table S1.1.

### Data preprocessing and identification of differentially expressed genes

To prioritise gene candidates with a possible relevance for the salinity response in the context of the subsequent expression study, we used publicly available RNAseq datasets from two sorghum genotypes with contrasting drought tolerance as template: the drought sensitive (DS) genotype IS20351, and the drought tolerant (DT) genotype IS22330 (accession number GSE80699)^61^. Read quality was checked twice before and after trimming (https://www.bioinformatics.babraham.ac.uk/projects/fastqc/) using Trimmomatic v0.39^62^. Subsequently, clean and trimmed reads were used for transcript quantification using the Salmon software in the non-alignment-based mode^63^. All samples were then mapped to the sorghum transcriptome (*Sbicolor_454_v3.1.1.transcript_primaryTranscriptOnly.fa;*https://phytozomenext.jgi.doe.gov/), with a mapping rate exceeding 84.99% (Supplemental Table S2). By using the DESeq2 pipeline^64^, differentially expressed genes (DEGs) responsive to salinity were identified by comparing the stressed and the non-stressed condition within each of the two genotypes, as well as comparing the two control conditions, and the two salt-stressed conditions between the two genotypes using default parameters (Supplemental Table S3, and S4). The same pipeline was also used to assess the quality of the RNA-seq data.

### Cloning and analysing the promoters of ***pSbSWEET13***, ***pSbSUT2***, and ***pSbSUT6***

Genomic DNA was extracted using the cetyltrimethylammonium bromide (CTAB) protocol^65^ from the leaves of Della and Razinieh and used as a template. Upstream promoter sequences of *SbSWEET13*, *SbSUT2*, and *SbSUT6* genes were amplified from the extracted genomic DNA of both genotypes, using Q5^®^ High-Fidelity DNA polymerase (NEB, Germany) based on oligonucleotide primers derived from the sorghum reference genome (BTx623). Details are given in Supplemental Table S1.2 The amplicons comprising the promoter fragments amplicons were obtained using 36 cycles of 10 s denaturation at 98 °C, 30 s annealing at 65 °C, and 120 s elongation at 72 °C. After elution from the gel and purification (Invisorb® Fragment CleanUp STRATEC), 2 µl (= 100 ng) of each amplicon was ligated into the pGEM^®^-T Easy Vector (*Promega* GmbH, Mannheim) and then transformed into E-coli DH5α for DNA sequencing (GATC Biotech, Cologne, Germany). Subsequently, the six promoter fragments were ligated into a GATEWAY version of the luciferase vector pLuc (Supplemental Figure S1), using the BP and LR recombination reactions (Invitrogen Corporation, Paisley, UK), respectively. After verification of inserts and transitional zones by sequencing (GATC Biotech, Cologne, Germany), putative regulatory elements were searched against the PlantCARE database (http://bioinformatics.psb.ugent.be/webtools/plantcare/html/), and aligned with the reference genome using the Clustalign Multiple Sequence Alignment tool (https://www.ebi.ac.uk/Tools/msa/clustalo/).

### Dual-luciferase promoter-reporter assay in the homologous system

To determine promoter activities in response to osmotic and salinity stress, as well as in response to MeJA and ABA as the most relevant phytohormones, we pursued the strategy of using a homologous readout system based on transient transfection with the promoter-reporter constructs. This process comprises the isolation of leaf protoplasts from sorghum, PEG-mediated transient transfection, and measuring promoter activity in response to the four inducers.

#### Generation of protoplasts

The methodology was developed based on a protocol by^62^ with some modifications. First, the caryopses of the sorghum genotype Della were surface sterilised and sown in magenta boxes (Sigma-Aldrich) containing solid growth medium made of 0.4% phytoagar medium mixed with 8% MS medium and 0.1% sucrose (Duchefa, The Netherlands). Seedlings were grown for 10 days in a culture room at 25 °C with a 12 h photoperiod at 120 µmol. m^–2^s^− 1^ light intensity and a 12 h dark period at 22 °C. Seedlings selected for uniformity were collected to excise the stem and leaf section with a sharp razor blade. Bundles of tissue from 30 individual plants were then cut further into strips of 3 mm width. The strips were pre-incubated in 10 mL of mannitol solution (500 mM), pH 5.7, in darkness for 30 min without shaking. Then, the mannitol solution was replaced with 10 mL of enzyme solution (500 mM mannitol, 0.6% cellulose, 0.375% macerozyme, 0.1% pectolyase, 0.1% BSA, and 0.1% polyvinylpyrrolidone K30), for 4:30 h in the dark at 26 °C under mild agitation shaking on an orbital shaker at 40 rpm. Then, an equal volume of W5 solution (154 mM NaCl, 125 mM CaCl_2_, 5 mM KCl, and 2 mM MES, pH 5.7) was added, gently mixed, and the incubation continued for 1 h at slightly more vigorous shaking (80 rpm). The mixed solution containing protoplasts was then filtered through a 70-nm nylon mesh (Corning^®^ cell strainer, REF 431751) into a 50 mL tube and centrifuged (Universal 320R, Andreas Hettich GmbH & Co. KG, Tuttlingen) at 1500 rpm for 3 min to collect the protoplasts. The isolated protoplasts were suspended in 300 µL of MMG suspension solution (0.4 M mannitol, 15 mM MgCl_2_, 4 mM MES, pH 5.7), and examined under Axioimager Z1 Apotome for completeness of the digest and the viability of the protoplasts (Supplemental Figure S2). Every solution in the process was sterilised by a 0.22 μm filter (Rotilabo^®^-SYRINGE FILTERS, PVDF, sterile, Carl Roth GmbH & Co. KG, Karlsruhe) before use.

#### Protoplast transfection

PEG-transfection was carried out as described in^66^ with minor changes. Aliquots of 300 µL protoplast suspension (1–2. 10^6^ cells/mL) were mixed with 75 µl (20 µg/mL) of the respective construct comprising the promoter of interest driving a firefly luciferase reporter, and 75 µl (20 µg/mL) of the plasmid pRLUC in a 2-mL reaction tube. This plasmid provides constitutive expression of the red Renilla luciferase under control of the constitutive Cauliflower Mosaic Virus (CaMV) 35 S promoter (Supplemental Figure S3) and allows normalisation for differences in transformation efficiency^67^. For transfection, 300 µL of 40% PEG 4000, complemented with 0.1 M CaCl_2_, and 0.4 M mannitol, pH 5.7 were added immediately and mixed gently by tilting the tube topover. The suspension was then incubated for 20 min at 26 °C, before adding 650 µL of W5 medium to dilute the PEG. The protoplasts were again collected by centrifugation (Universal 320R, Andreas Hettich GmbH & Co. KG, Tuttlingen) at 500× g for 5 min and suspended in 650 µL of incubation buffer (0.5 M mannitol, 4 mM KCl, 4 mM MES, pH 5.7) and allowed to equilibrate at 26 °C for 48 h.

#### Measuring promoter activation

After equilibration for 48 h, the protoplasts were ready to measure induction by different conditions. In addition to osmotic stress (25% PEG 6000) and salinity (200 mM NaCl), the responses to 25 µM ABA and 50 µM MeJA, as crucial hormonal regulators of the stress response were assessed. The inducers were allowed to act for 1 h. Then, cells were harvested by centrifugation (microcentrifuge, VWR Microstar 17, VWR International GmbH, Darmstadt) with 8000 g for 1 min. After discarding 650 µl of the supernatant, the sedimented protoplasts were lysed by adding 100 µl of 2× passive lysis buffer (PLB, Promega, Madison, Wl) to the cells on ice, and vortexing for 30 s, followed by shaking for 10 min at 500 rpm. After removing cellular debris by centrifugation for 1 min at 10.000 g, luciferase activities were measured in the supernatants using the dual-luciferase reporter assay system (PJK, Kleinblittersdorf, Germany) according to^68^. In brief, for each lysate supernatant (20 ml), 50 µl of each Beetle Juice and Renilla Glow Juice were added. The emitted bioluminescence was then measured with a luminometer (lumat LB9507, Berthold Technologies, Bad Wildbad, Germany). The relative luciferase activity was then normalised by the ratio between the firefly and Renilla (control for transfection efficiency) luciferase activity. Each data point represents mean and standard error from three independent transfection experiments.

## Electronic supplementary material

Below is the link to the electronic supplementary material.

Supplementary Material 1.

Supplementary Material 2.

Supplementary Material 3.

Supplementary Material 4.

Supplementary Material 5.

Supplementary Material 6.

Supplementary Material 7.

Supplementary Material 8.

Supplementary Material 9.

Supplementary Material 10.

Supplementary Material 11.

Supplementary Material 12.

Supplementary Material 13.

Supplementary Material 14.

Supplementary Material 15.

Supplementary Material 16.

Supplementary Material 17.

Supplementary Material 18.

Supplementary Material 19.

Supplementary Material 20.

Supplementary Material 21.

## Data Availability

All data supporting the findings of this study are available within the paper and within its supplementary materials published online. Any other data are available from the corresponding author EA upon request.
