# Supplementary figures and images for: Salt stress-induced remodeling of sugar transport: a role for promoter alleles of SWEET13
Source: Sci Rep. 2025 Mar 4;15:7580. doi: 10.1038/s41598-025-90432-2 (PMC11880500; doi:10.1038/s41598-025-90432-2)

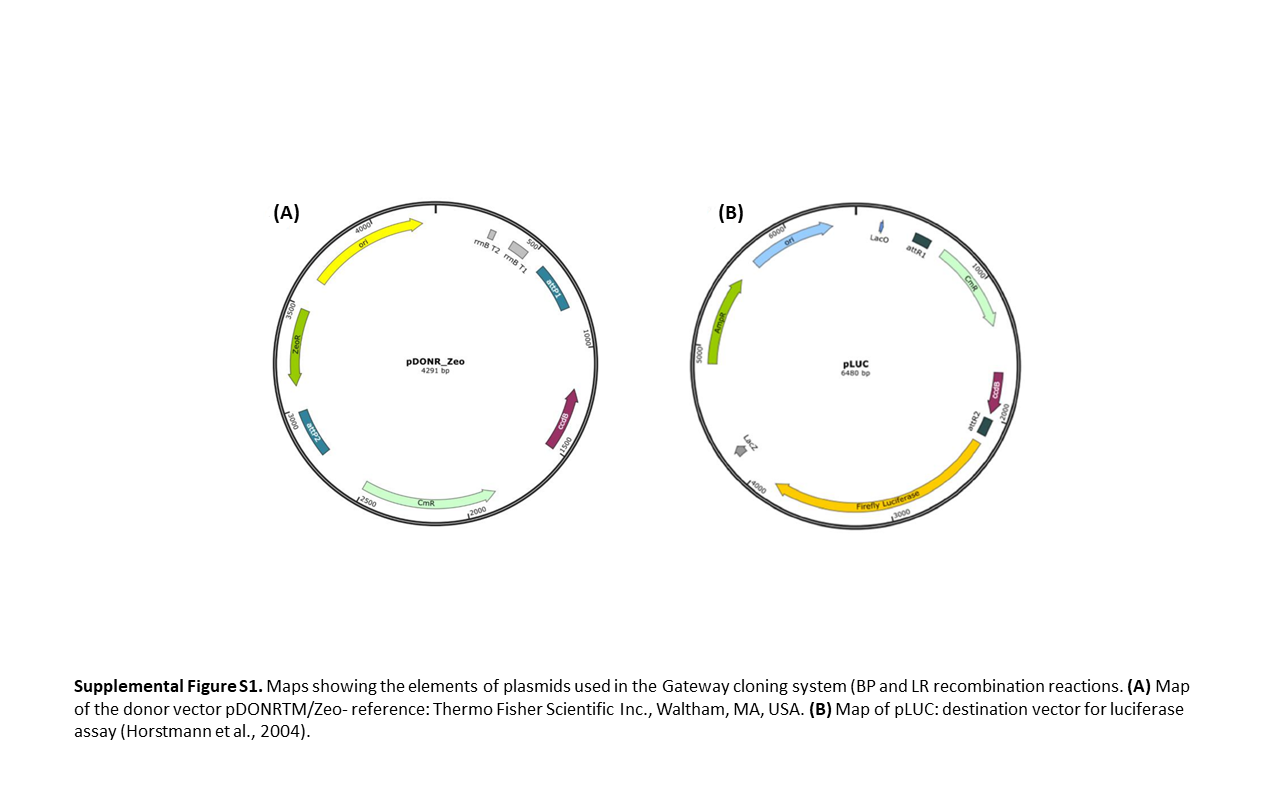

Supplement: Supplementary file 1 — Supplementary Material 1. [file 41598_2025_90432_MOESM1_ESM.tif]

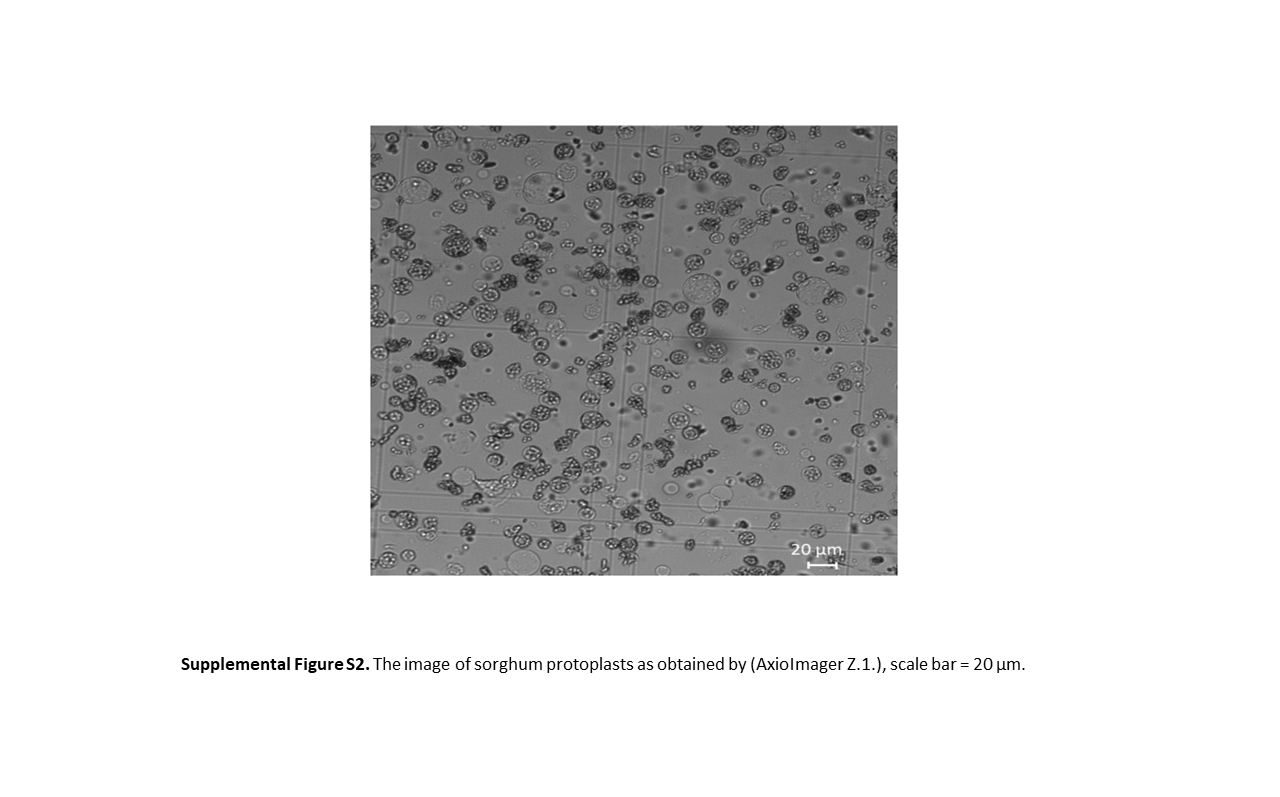

Supplement: Supplementary file 2 — Supplementary Material 2. [file 41598_2025_90432_MOESM2_ESM.tif]

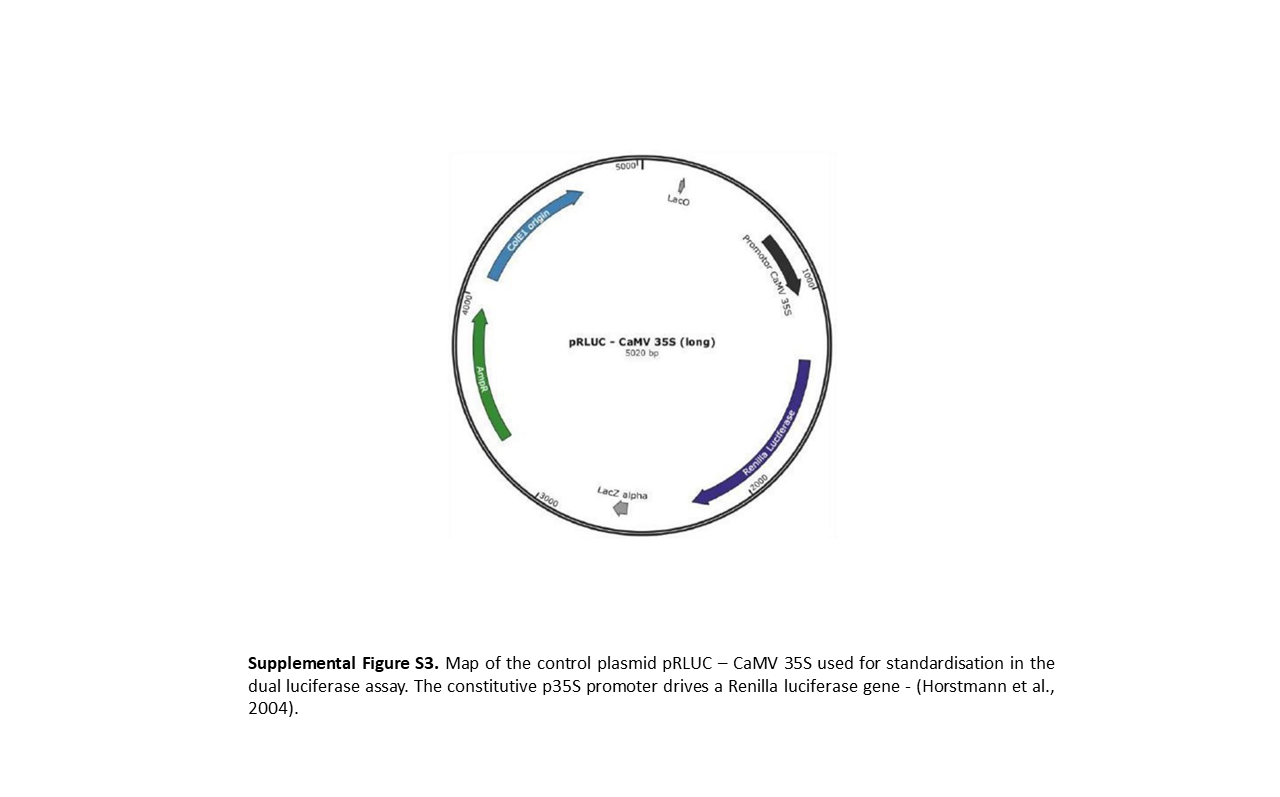

Supplement: Supplementary file 3 — Supplementary Material 3. [file 41598_2025_90432_MOESM3_ESM.tif]

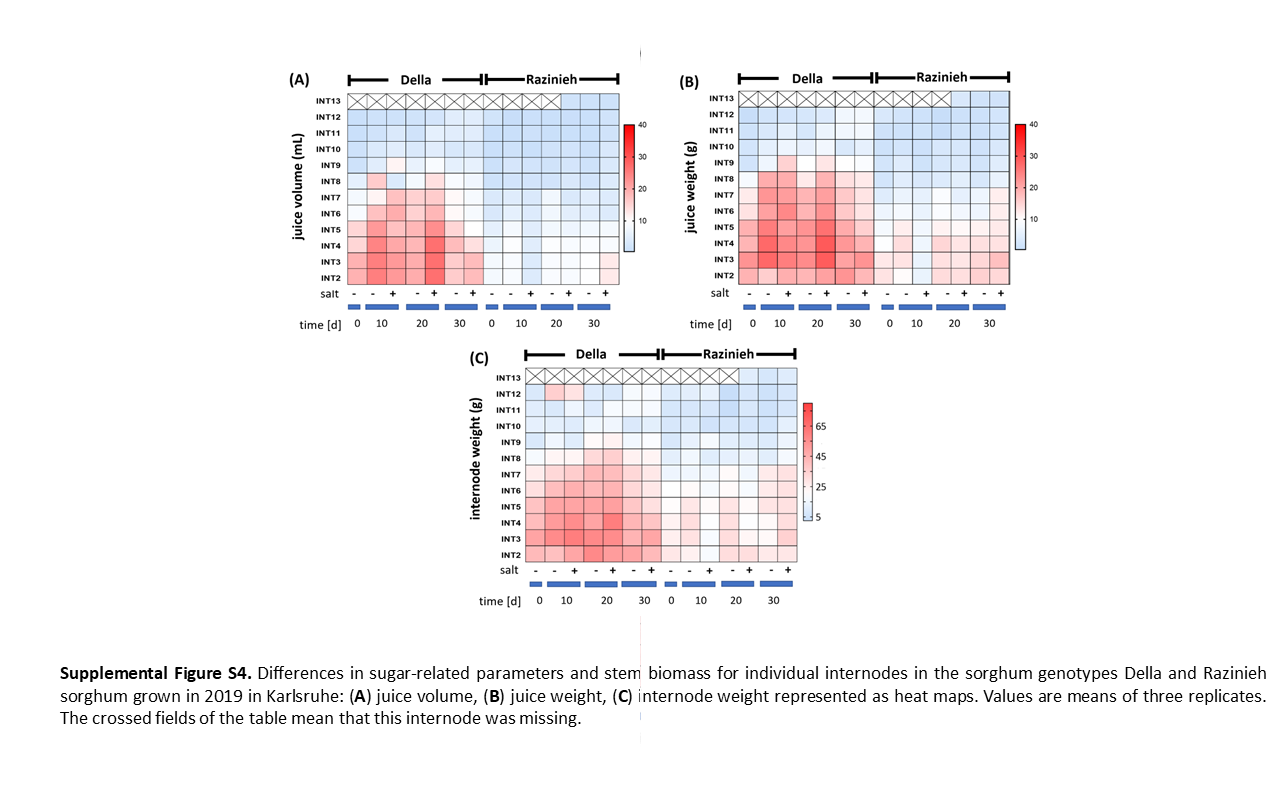

Supplement: Supplementary file 4 — Supplementary Material 4. [file 41598_2025_90432_MOESM4_ESM.tif]

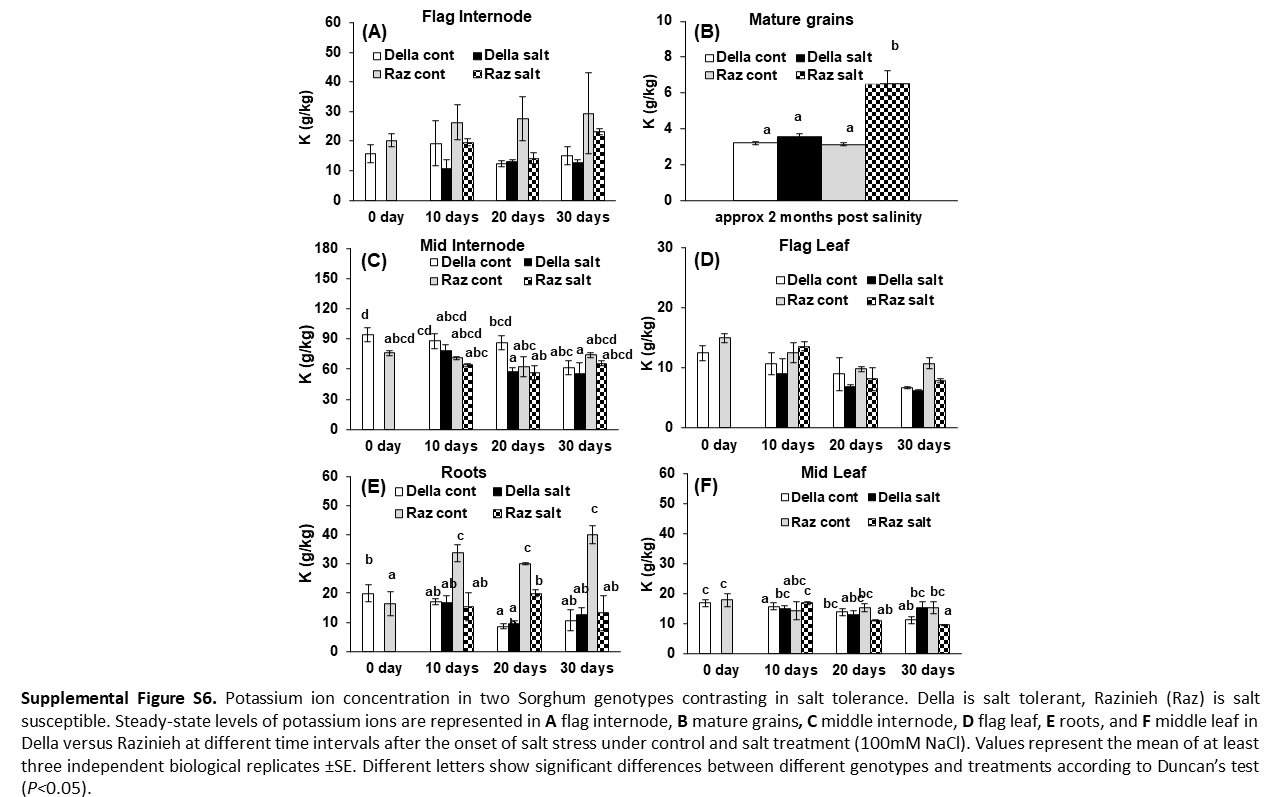

Supplement: Supplementary file 5 — Supplementary Material 5. [file 41598_2025_90432_MOESM5_ESM.tif]

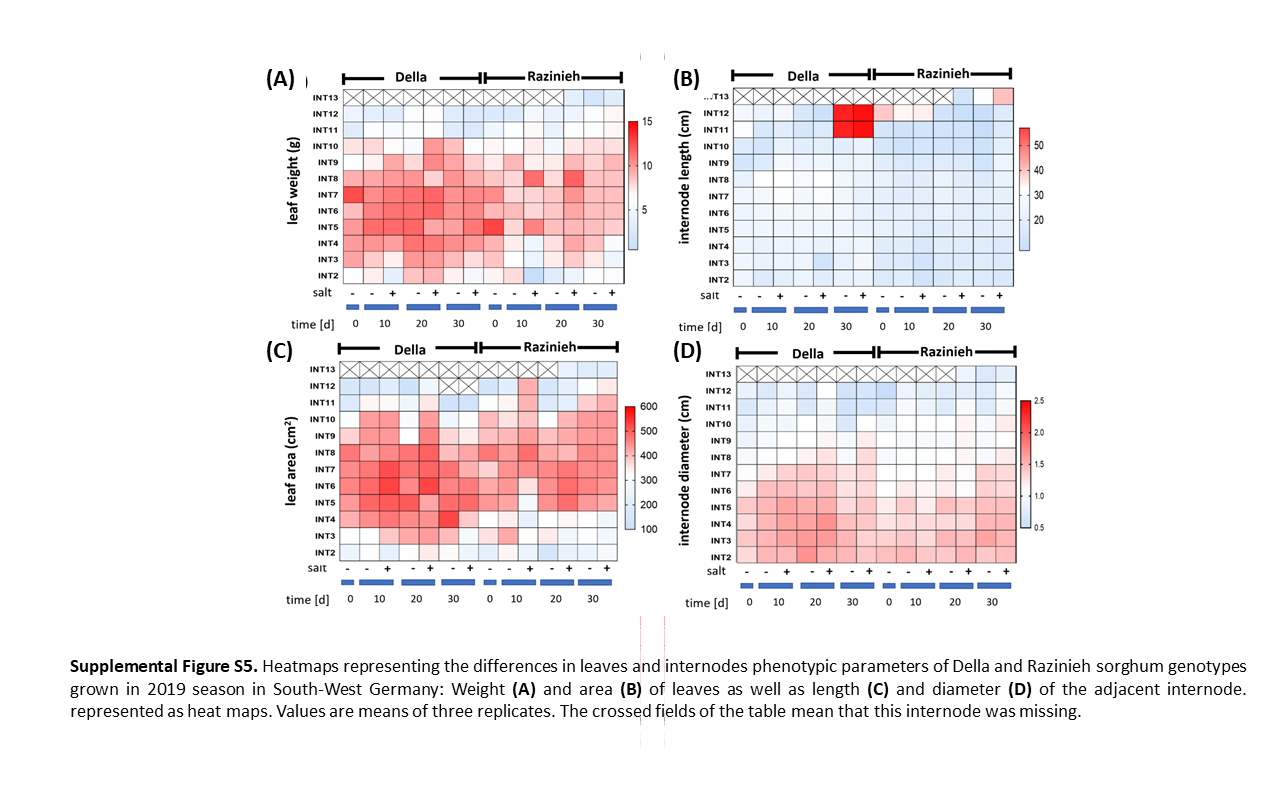

Supplement: Supplementary file 6 — Supplementary Material 6. [file 41598_2025_90432_MOESM6_ESM.tif]

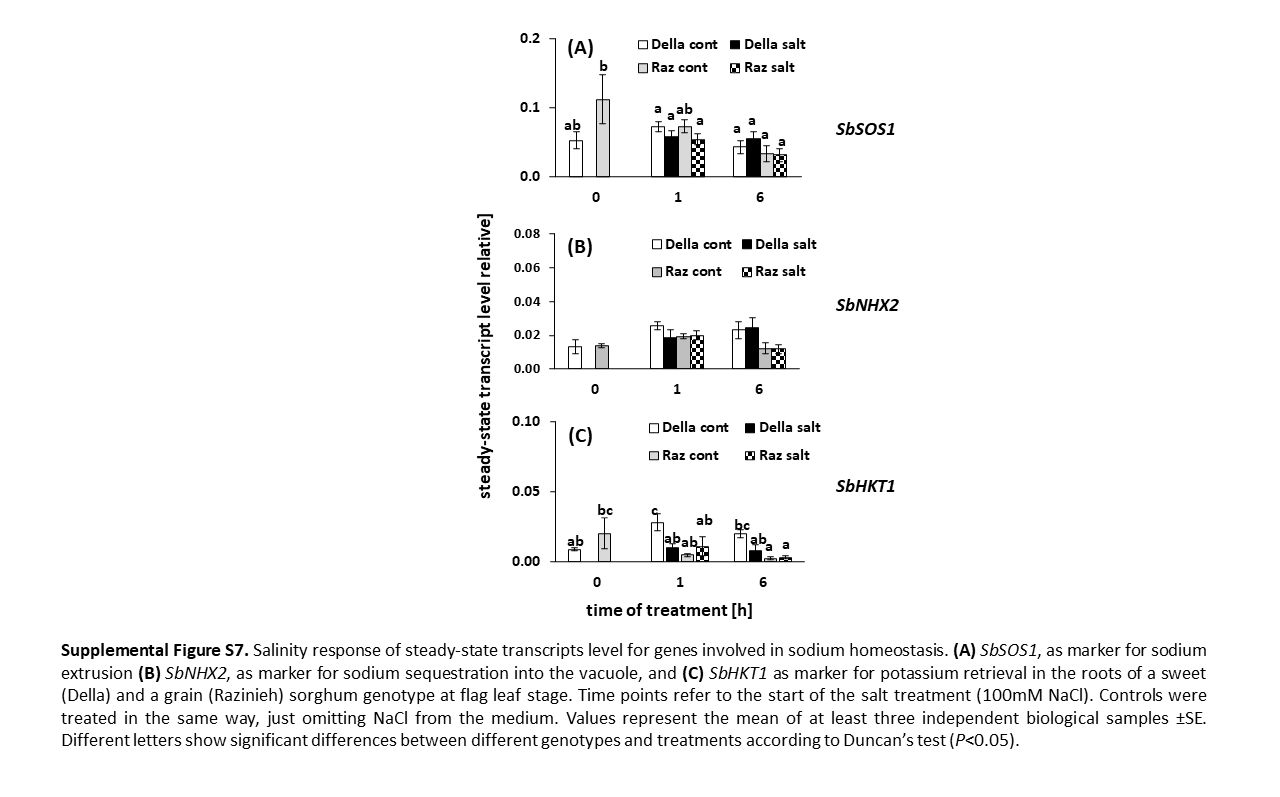

Supplement: Supplementary file 7 — Supplementary Material 7. [file 41598_2025_90432_MOESM7_ESM.tif]

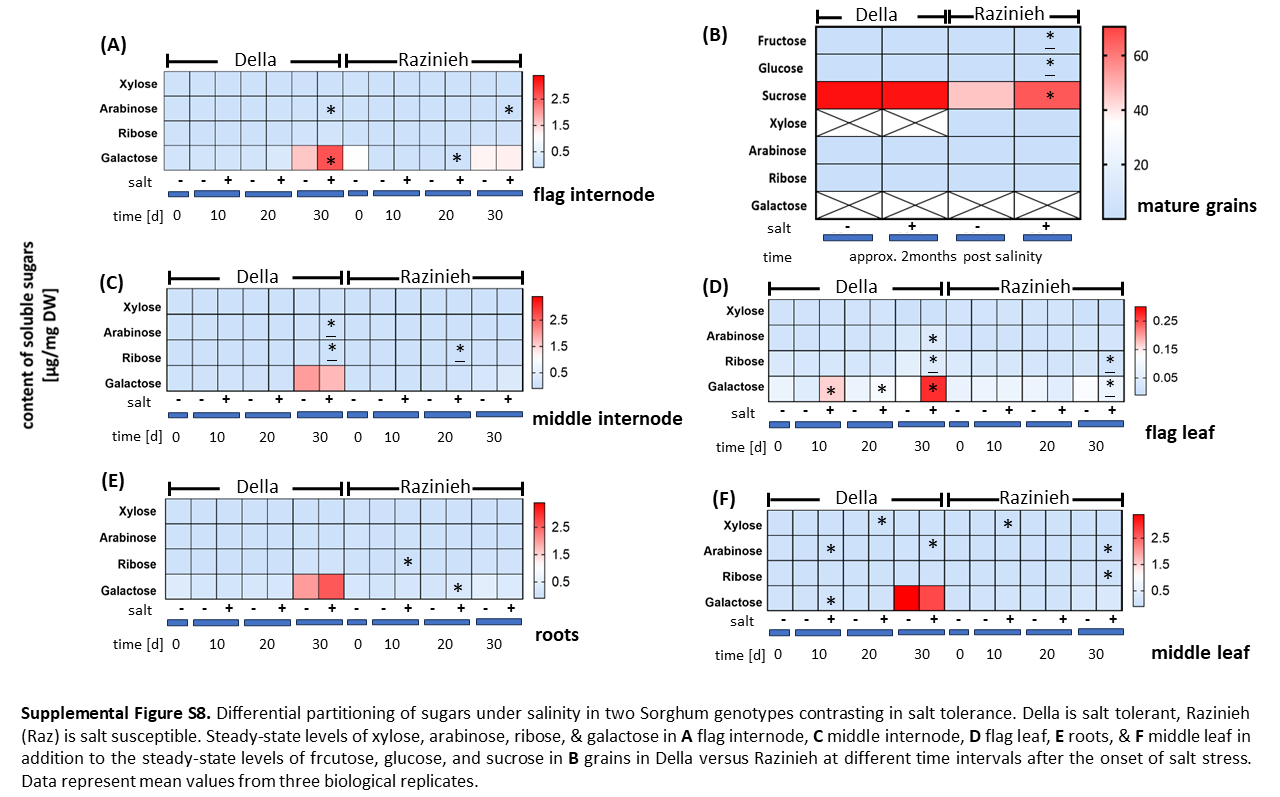

Supplement: Supplementary file 8 — Supplementary Material 8. [file 41598_2025_90432_MOESM8_ESM.tif]

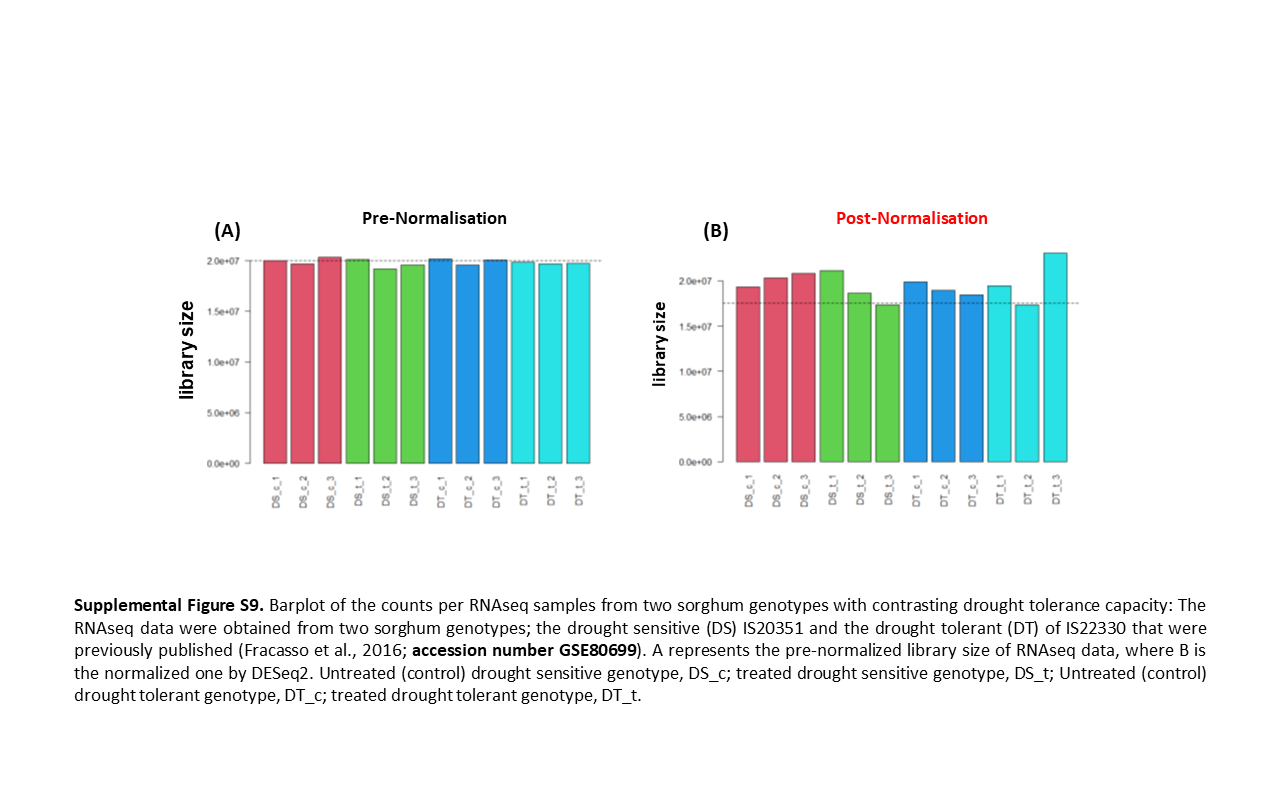

Supplement: Supplementary file 9 — Supplementary Material 9. [file 41598_2025_90432_MOESM9_ESM.tif]

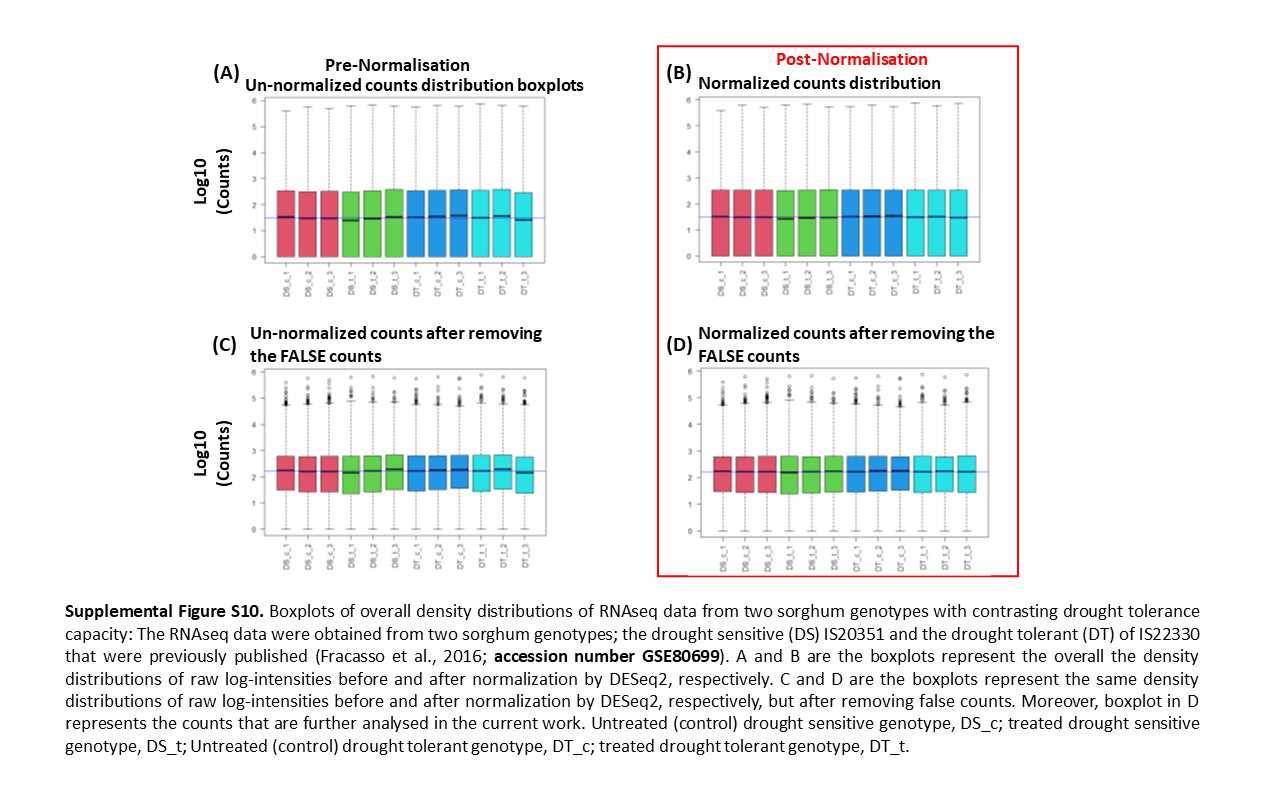

Supplement: Supplementary file 10 — Supplementary Material 10. [file 41598_2025_90432_MOESM10_ESM.tif]

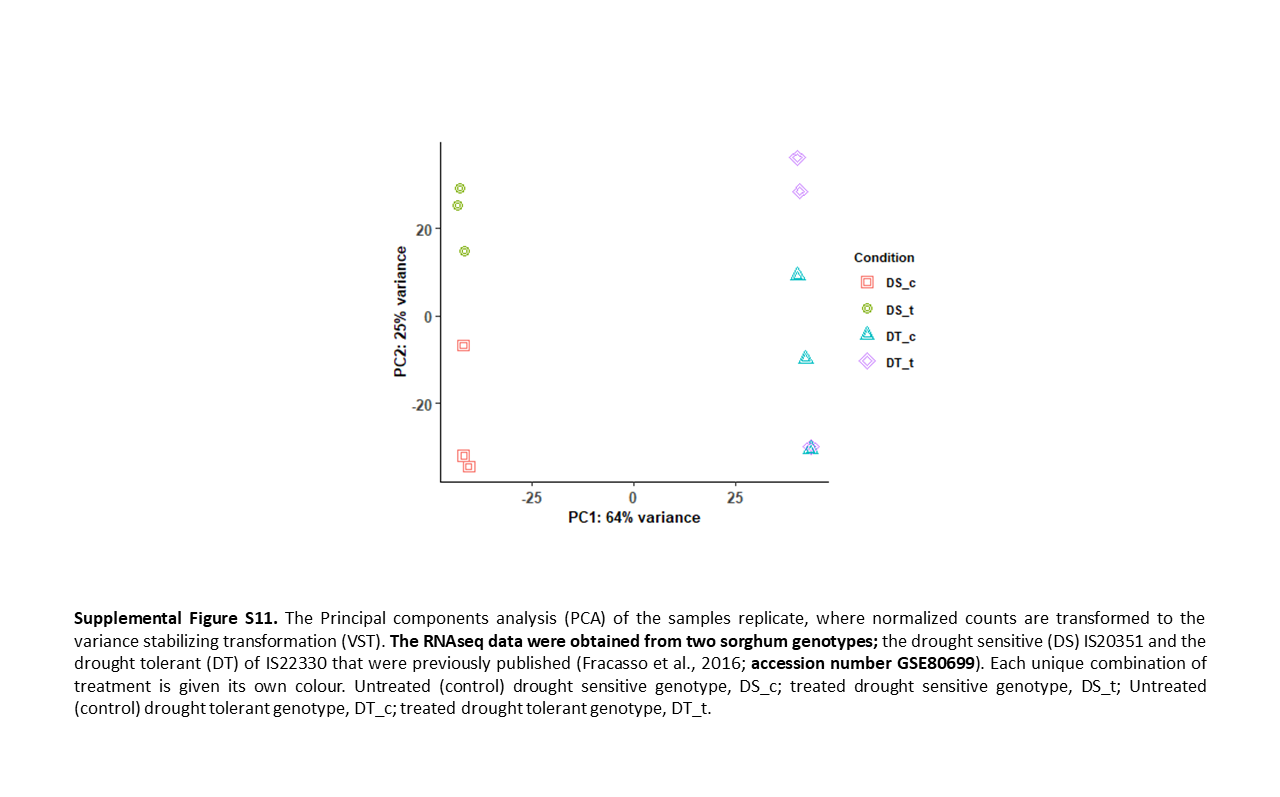

Supplement: Supplementary file 11 — Supplementary Material 11. [file 41598_2025_90432_MOESM11_ESM.tif]

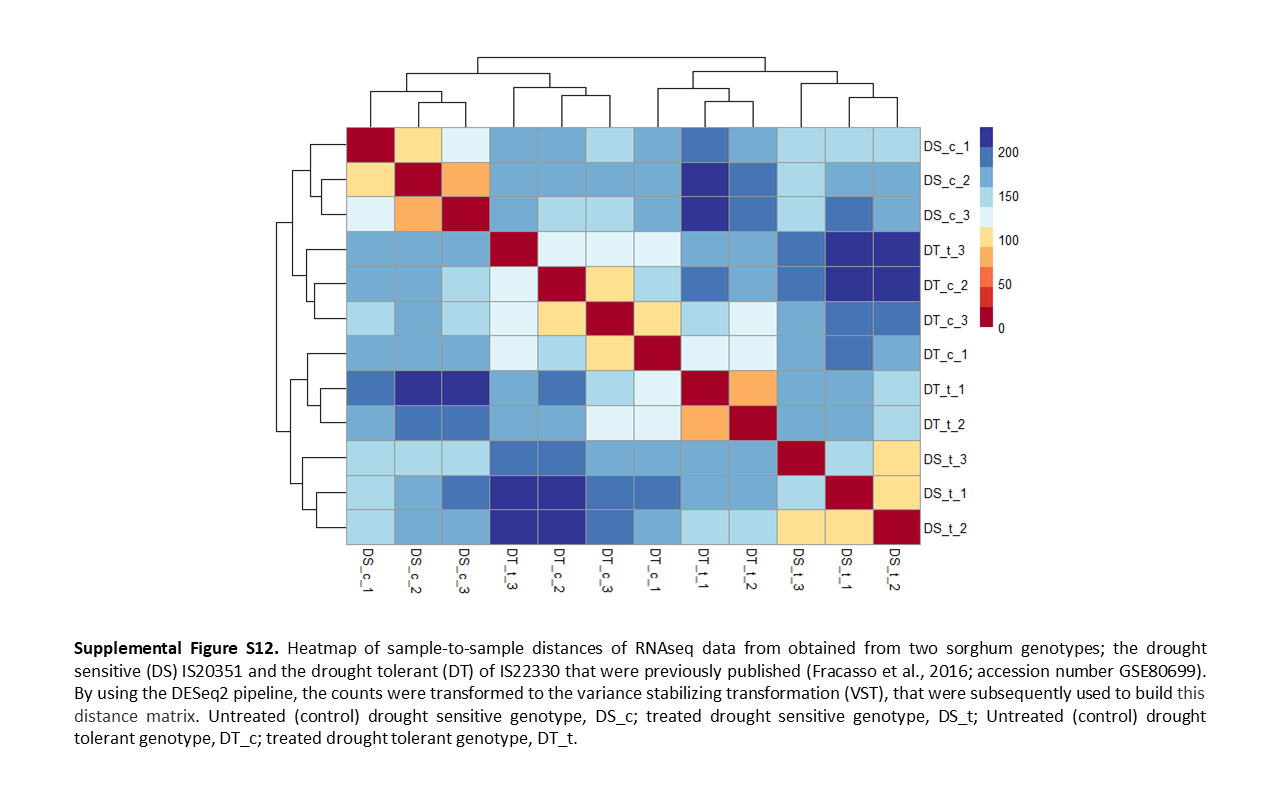

Supplement: Supplementary file 12 — Supplementary Material 12. [file 41598_2025_90432_MOESM12_ESM.tif]
