# Supplementary material for: Salt stress-induced remodeling of sugar transport: a role for promoter alleles of SWEET13
Source: Sci Rep. 2025 Mar 4;15:7580. doi: 10.1038/s41598-025-90432-2 (PMC11880500; doi:10.1038/s41598-025-90432-2)
Supplement: Supplementary file 13 — Supplementary Material 13. [file 41598_2025_90432_MOESM13_ESM.docx]

**Supplementary Table S1.1. List of primers used for qPCR**

| **Gene name** | **Accession No.** | **Forward(5’-3’ prime)** | **Reverse(5’-3’ prime)** |
| --- | --- | --- | --- |
| ***SbUbiquitin*** | XM_002439293.2 | CAAGGAGTGCCCCAACAC | TGGTAGGCGGGTAAAGCAAA |
| ***SbGAPDH*** | XM_021449348.1 | AGGGTATCATGGGCTACGTG | AGTTGTCGTTCAGGGCAATC |
| ***SbSPS1*** | XM_002458946.2 | ACGCGACAAGACTTCAGGTT | ATCGGTATCGCCATGTTCCC |
| ***SbSPS4*** | XM_002441477.2 | TCCTGTTTTGGCTTCTCGCT | TTTGTGCACGCCTCCAAGTA |
| ***SbSUS3*** | XM_002465258.2 | AAGCTTGAGAGGCGGGAGAC | TGAGCAAGCTACGAGCACCA |
| ***SbSUS4*** | XM_021449494.1 | TGCTGCCCCCTCTATTTATTGG | GAGGAGAAGGTGGCACCAAG |
| ***SbCINV1*** | XM_002453920.2 | AGGCGATGTTGAAGCCTGTT | CCCGGTTTGGAAGTCAAGGA |
| ***SbCINV2*** | XM_002452587.2 | TGCTTTCCGAGTTCCTACTGG | TCCTTATTTCCCCGACCAAACT |
| ***SbSUT1*** | KP685701.1 | GTGCTCCTGTAATCTTTGTGTCC | ACTATACTGCACATTGATTGATCG |
| ***SbSUT2*** | KY287230.1 | GCACATGCATTGAATGAACC | TTCGCATTTGGAAATTCCTC |
| ***SbSUT4*** | KY287232.1 | ATGCAATGGCTGCTAGTCGT | CACCGCCGAACATTTGATCC |
| ***SbSUT5*** | XM_002454013.2 | CCCGTAGTGTTGCGGAGTC | CCAATGGATCGGAAAATAAAG |
| ***SbSUT6*** | KY287234.1 | CTCCTCTTCTGCTCCGTCGC | GAACACCATGAGGTTGCTGA |
| ***SWEET6*** | XM_002455874.2 | CGCTCATCCGCTTCGACC | GGGCAGCTCCACGTTCTT |
| ***SbSWEET13*** | XM_002442074.2 | CTCTCCATCGCAAGCAAGCA | GCTAAGGGTTGGATAAACGGG |
| ***SbbZIP-TF-TRAB1*** | XM_021454381.1 | CAGTGGTGCTTGGGATCTGT | ACTACATAAGCGGCGGAAGG |
| ***SbNHX2*** | XM_002461123.2 | TTGGCACCAAGAGACGAACA | CGCCATTGCTAACGCATCAT |
| ***SbHKT1*** | XM_002457691.2 | GCAATGTGGGATTCAGCACC | CTTGAGCCTGCCGTAGAACA |
| ***SbSOS1*** | [XM_002443629.2](https://www.ncbi.nlm.nih.gov/entrez/viewer.fcgi?db=nucleotide&id=1205974562) | AGTGGCAGCTCTACCTCTCA | TCGTCGCTTGAGTTGTCCTC |

**Supplementary Table S1.2. List of primers used for promoters amplifying, sequencing, and GATEWAYcloning**

| **Gene name** | **Accession No.** | **Forward(5’-3’ prime)** | **Reverse(5’-3’ prime)** |
| --- | --- | --- | --- |
| ***pSbSWEET13*** | XM_002442074.2 | CGGTGAGGAGTCAAACACAA | AGGGGTGCTGCAGAGATAGG |
| ***pSbSUT2*** | KY287230.1 | CTCTCTGCCACGGTGAAGTC | GAGGCTGACGAGCTCCATCT |
| ***pSbSUT6*** | KY287234.1 | GTCAGTTCTAGCTCCACTGTCA | GAGACCATGCAGGCCAAGAA |
| ***attB-pSbSWEET13*** | XM_002442074.2 | GGGGACCACTTTGTACAAGAAAGCTGGGTCAGGGGTGCTGCAGAGATAGG | GGGGACCACTTTGTACAAGAAAGCTGGGTCAGGGGTGCTGCAGAGATAGG |
| ***attB-pSbSUT2*** | KY287230.1 | GGGGACAAGTTTGTACAAAAAAGCAGGCTTCCTCTCTGCCACGGTGAAGTC | GGGGACCACTTTGTACAAGAAAGCTGGGTCGAGGCTGACGAGCTCCATCT |
| ***attB-pSbSUT6*** | KY287234.1 | GGGGACAAGTTTGTACAAAAAAGCAGGCTTCGTCAGTTCTAGCTCCACTGTCA | GGGGACCACTTTGTACAAGAAAGCTGGGTCGAGACCATGCAGGCCAAGAA |
