# Supplementary material for: Salt stress-induced remodeling of sugar transport: a role for promoter alleles of SWEET13
Source: Sci Rep. 2025 Mar 4;15:7580. doi: 10.1038/s41598-025-90432-2 (PMC11880500; doi:10.1038/s41598-025-90432-2)
Supplement: Supplementary file 16 — Supplementary Material 16. [file 41598_2025_90432_MOESM16_ESM.doc]

**Supplementary Table S9.** The alignment and *cis*-elements for SWEET13, SUT2, and SUT6 promoters of Della and Razinieh sorghum genotypes, compared to the reference genome BTX623.

**SWEET13 promoter**

Della CGGTGAGGAGTCAAACACAAAGTTTTATAATTGCTCAACATATGCCGTTTTATTTGAGAA 60

Razinieh gtgcaggcagtcaaaCACAAAGTTTTATAATTGCTCAACATATG--------------CC 46

BTX623 CGGTGAGGAGTCAAACACAAAGTTTTATAATTGCTCAACATATGCCGTTTTATTTGAGAA 60

* * ************************************

Dehydration responsive element(DRE)

Della AAGTGTGGTGTTCTGATGCTGACCAGTGGCACACAGCCACAAGATTAAAAACAGACCGAC 120

Razinieh GTTTGtGgTGTTCTGATGCTGACCAGTGGCACaCAgcCACAAGATTAAAAACAGACCGAC 106

BTX623 AAGTGTGGTGTTCTGATGCTGACCAGTGGCACACAGCCACAAGATTAAAAACAGACCGAC 120

*********************************************************

Della AGATTCAAGACAATATATCATTAAATCAGTTCGTAGATTAGATCGTCGCCTATGTTCATA 180

Razinieh AGATTCAAGACAATATATCATTAAATCAGTTCGTAGATTAGATCGTCGCCTATGTTCATA 166

BTX623 AGATTCAAGACAATATATCATTAAATCAGTTCGTAGATTAGATCGTCGCCTATGTTCATA 180

************************************************************

Della GGAGAAAATATTCTTGGCCACCCGTGCCAACTGTTTGGAGACCACCAAAACCAAGTCTTG 240

Razinieh GGAGAAAATATTCTTGGCCACCCGTGCCAACTGTTTGGAGACCACCAAAACCAAGTCTTG 226

BTX623 GGAGAAAATATTCTTGGCCACCCGTGCCAACTGTTTGGAGACCACCAAAACCAAGTCTTG 240

************************************************************

Della AGAAACCACCTTATATAGAATAGCACAAGTACATAGTGAATGGATAGCTAGATGCGCGTA 300

Razinieh AGAAACCACCTTATATAGAATAGCACAAGTACATAGTGAATGGATAGCTAGATGCGCGTA 286

BTX623 AGAAACCACCTTATATAGAATAGCACAAGTACATAGTGAATGGATAGCTAGATGCGCGTA 300

************************************************************

Della GCAAGTCTTGTGCAATCAGGTGTCTAATTGTCTTAAGCCACACAATTTACTAACTTGCCT 360

Razinieh GCAAGTCTTGTGcaATCAGGTGTCTAATTGTCTTAAGCCACACAATTTACTAACTTGCCT 346

BTX623 GCAAGTCTTGTGCAATCAGGTGTCTAATTGTCTTAAGCCACACAATTTACTAACTTGCCT 360

************************************************************

Della GATGTGCTTGGCAAGATTATCCTTGGCCAATACAACTCCTCTTTGTAAGTACCATAAAAA 420

Razinieh GATGTGCTTGGCAAGATTATCCTTGGCCAATACAACTCCTCTTTGTAAGTACCATAAAAA 406

BTX623 GATGTGCTTGGCAAGATTATCCTTGGCCAATACAACTCCTCTTTGTAAGTACCATAAAAA 420

************************************************************

Della GATTTTGATTTTTTGATGGGGCCTTCAGTTTCCATAAACTTTTGTTGAAATTGGTATCGA 480

Razinieh GATTTTGATTTTTTGATGGGGCCTTCAGTTTCCATAAACTTTTGTTGAAATTGGTATCGA 466

BTX623 GATTTTGATTTTTTGATGGGGCCTTCAGTTTCCATAAACTTTTGTTGAAATTGGTATCGA 480

************************************************************

Della TTAACATCACTGAGAATTTTCCATTTGGGTGTAAATTCCAATGGAAACATGTAGGTCCTG 540

Razinieh TTAACATCACTGAGAATTTTCCATTTGGGTGTAAATTCCAATGGAAACATGTAGGTCCTG 526

BTX623 TTAACATCACTGAGAATTTTCCATTTGGGTGTAAATTCCAATGGAAACATGTAGGTCCTG 540

************************************************************

Della TGACAATTGTATCTATAACTCTGATAAAGCAAAACCCCACTAGCTATTTCTCTTGACATT 600

Razinieh TGACAATCGTATCTATAACTCTGATAAAGCAAAACCCCACTAGCTATTTCTCTTGACATT 586

BTX623 TGACAATTGTATCTATAACTCTGATAAAGCAAAACCCCACTAGCTATTTCTCTTGACATT 600

******* ****************************************************

Della TGAGGCGTCCACCTCAGCAGTCCATTATGATTTGCCACTATCAATTTCTCTTGACATGCA 660

Razinieh TGAGGCGTCCACCTCAGCACTCCATTATGATTTGCCACTATCAATTTCTCTTGACATGCA 646

BTX623 TGAGGCGTCCACCTCAGCAGTCCATTATGATTTGCCACTATCAATTTCTCTTGACATGCA 660

******************* ****************************************

Della CCTCAGTAGCCCACTATCATTTGTAATTAAAGTTCACTAGCAC-AAGAATTATGATATCC 719

Razinieh CCTCAGCAGCCCACTATCATTTGTAATTAAAGTTCACTAGCACCAAGAATTAtgATATCC 706

BTX623 CCTCAGTAGCCCACTATCATTTGTAATTAAAGTTCACTAGCAC-AAGAATTATGATATCC 719

****** ************************************ ****************

MeJA-responsive element

Della ACGTCAGCCAAATATATTATCCACAATGGATACAATCTTTAAGATTATACTTTACACCCG 779

Razinieh ACATCAGCCAAATATATTATCCACTATGGATACAATCTTTAAGATTATACTTTACACCCG 766

BTX623 ACGTCAGCCAAATATATTATCCACAATGGATACAATCTTTAAGATTATACTTTACACCCG 779

** ********************* ***********************************

Della CGGCAATGTGTGTGGGGAATCCTTCTAGTATTAGTAATGCGTGGTAAATGGTTGTTCCAA 839

Razinieh CGGCAATGTGTGTGGGGAATCCTTCTAGTATTAGTAATGCGTGGTAAATGGTTGTTCCAA 826

BTX623 CGGCAATGTGTGTGGGGAATCCTTCTAGTATTAGTAATGCGTGGTAAATGGTTGTTCCAA 839

************************************************************

Della GCTACTTAGGATTTGTATAATATGCTTATTGTCCTCCCAAAACCTCAATTGCATACTGTT 899

Razinieh GCTACTTAGGATTTGTATAATATGCTTATTGTCCTCCCAAAACCTCAATTGCATACTGTT 886

BTX623 GCTACTTAGGATTTGTATAATATGCTTATTGTCCTCCCAAAACCTCAATTGCATACTGTT 899

************************************************************

Della ATAAAGACTTGGATACTGTTCTTACAAAGTTGTGTTTCGAAGCCGTTTGTCCTTCTAGAA 959

Razinieh ATAAAGACTTGGATACTGTTCTTACAAAGTTGTGTTTCGAAGCCGTTTGTCCTTCTAGAA 946

BTX623 ATAAAGACTTGGATACTGTTCTTACAAAGTTGTGTTTCGAAGCCGTTTGTCCTTCTAGAA 959

************************************************************

Della TCTCAATTGCCAAATCATAAATAAATTATTTTATTGTTAGGTTACAAGATATAACTTGGT 1019

Razinieh TCTCAATTGCCAAATCATAAATAAATTATTTTATTGTTAGGTTACAAGATATAACTTGGT 1006

BTX623 TCTCAATTGCCAAATCATAAATAAATTATTTTATTGTTAGGTTACAAGATATAACTTGGT 1019

************************************************************

Della ACTAAAATCACAAATTGCCAATGGAATGAAGTAAATGTGTCTTTGGAGTCGAAAGTTGTG 1079

Razinieh ACTAAAATCACAAATTGCCAATGGAATGAAGTAAATGTGTCTTTGGAGTCGAAAGTTGTG 1066

BTX623 ACTAAAATCACAAATTGCCAATGGAATGAAGTAAATGTGTCTTTGGAGTCGAAAGTTGTG 1079

************************************************************

Della AAGATCAGTTTTGCTGTTGTGTGGCGGCTAGCTCCTACTCGATCTTAATTAGTTAATGAT 1139

Razinieh AAGATCAGTTTTGCTGTTGTGTGGCGGCTAGCTCCTACTCGATCTTAATTAGTTAATGAT 1126

BTX623 AAGATCAGTTTTGCTGTTGTGTGGCGGCTAGCTCCTACTCGATCTTAATTAGTTAATGAT 1139

************************************************************

Della GTATGCGAATGAAGCAGGTTGGAGAGTTCCACGGGCAGTGGATAACAATTCGGAGTGTAC 1199

Razinieh GTATGCGAATGAAGCAGGTTGGAGAGTTCCACGGGCAGTGGATAACAATTCGGAGTGTAC 1186

BTX623 GTATGCGAATGAAGCAGGTTGGAGAGTTCCACGGGCAGTGGATAACAATTCGGAGTGTAC 1199

************************************************************

Della TACCCCAGCACTACTAGCCCATATATTGTGCTCATAAAGCTTGCACAAAGCTAACTCTTT 1259

Razinieh TACCCCAGCACTACTAGCCCATATATTGTGCTCATAAAGCTTGCACAAAGCTAACTCTTT 1246

BTX623 TACCCCAGCACTACTAGCCCATATATTGTGCTCATAAAGCTTGCACAAAGCTAACTCTTT 1259

************************************************************

Della ATGCAACGACCATATTATCATCTTTATTAGTATATGTCAGCTTATAAAAATACAATTATT 1319

Razinieh ATGCAACGACCATATTATCATCTTTATTAGTATATGTCAGCTTATAAAAATACAATTATT 1306

BTX623 ATGCAACGACCATATTATCATCTTTATTAGTATATGTCAGCTTATAAAAATACAATTATT 1319

************************************************************

Della CATCATGTCCAGAAATTGTCAGTGGAAAGTATCTTTGAATTATATTCCATACTTAATTGT 1379

Razinieh CATCATGTCCAGAAATTGTCAGTGGAAAGTATCTTTGAATTATATTCCATACTTAATTGT 1366

BTX623 CATCATGTCCAGAAATTGTCAGTGGAAAGTATCTTTGAATTATATTCCATACTTAATTGT 1379

************************************************************

ABRE, ABA-responsive element

Della TTGGGGTTAGATGTGAGATTGGGGAGGCTATAGTGCACGCTGCAGACGTGTACACAATGT 1439

Razinieh TTGGGGTTAGATGTGAGATTGGGGAGGCTATAGTGCAGGCTGCAGACGTGTACACAATGT 1426

BTX623 TTGGGGTTAGATGTGAGATTGGGGAGGCTATAGTGCACGCTGCAGACGTGTACACAATGT 1439

************************************* **********************

Della CACAACCCACATGTATACCAAGGCATGCGTGCCACCTCCTATATAAAGCCCCCAACAGCC 1499

BTX623 CACAACCCACATGTATACCAAGGCATGCGTGCCACCTCCTATATAAAGCCCCCAACAGCC 1499

Razinieh CACAACCCACATGTATACCAAGGCATGCGTGCCACCTCCTATATAAAGCCCCCAACAGCC 1486

************************************************************

Della AGCGTATCATTGCCAGAGTTTCTGACAACAACTCAGCTGAGAACTCCTTGCAGAGCTCTT 1559

Razinieh AGCGTATCATTGCCAGAGTTTCTGACAACAACTCagCTGAGAACTCCTTGCAGAGCTCTT 1546

BTX623 AGCGTATCATTGCCAGAGTTTCTGACAACAACTCAGCTGAGAACTCCTTGCAGAGCTCTT 1559

************************************************************

Della CGATCTTGCCCATAGCACCTGCAACTGTTAGTTCAGTTGTGTCGCAATGGCAGGCCTATC 1619

Razinieh CGATCTTGCCCATAGCACCTGCAACTgttAGTTCAGTTGTGTCGCAaTGGCAGGCCTATC 1606

BTX623 CGATCTTGCCCATAGCACCTGCAACTGTTAGTTCAGTTGTGTCGCAATGGCAGGCCTATC 1619

************************************************************

Della TCTGC 1624

Razinieh TCTGC 1611

BTX623 TCTGC 1624

*****

**SUT2 promoter**

MeJA-responsive element

Della CTCcCTGCCACggTGAAGTCTTCTTGACGCAGGCAGCTAGaACCCCTAGCCAGCGCGGCG 60

Razinieh CTCtCTGcCACGGtGAAGTCTTCTTGACGCAGGCAGCTAGAACCCCTAGCCAGCGCGGCG 60

BTx623 CTCTCTGCCACGGTGAAGTCTTCTTGACGCAGGCAGCTGGAACCCCTAGCCAGCGCGGCG 60

*** ********************************** *********************

Della CTTGTCGGCGGCTTTGCCCCTCTGTGGCACTTGATCATCTTTGgtTATCCCCATCATCTT 120

Razinieh CTTGTCGGCGGCTTTGCCCCTCTGTGGCACTTGATCATCTTTGGTTATCACCATCATCTT 120

BTx623 CTTGTCGGCGGCTTTGCCCCTCTGTGGCACTTGATCATCTTTGGTTATCACCATCATCTT 120

************************************************* **********

Della CTTTTGCTCtATCACTTGGTATGTACCAACCTCATCTACACTTAGCATGAAGGTTAGTAC 180

Razinieh CTTTTGCTCTATCACTTGGTATGTACCAACCTCATCTACACTTAGCATGAAGGTTAGTAC 180

BTx623 CTTTTGCTCTATCACTTGGTATGTACCAACCTCATCTACACTTAGCATGAAGGTTAGTAC 180

************************************************************

Della TTAGGTTTCAtCAATTATCCAAAACCaTACTAGAACTTTCAATCTCATCCTTTTTGGTAA 240

Razinieh TTAGGTTTCATCAATTATCCAAAACCATACTAGAACTTTCAATCTCATCCTTTTTGGTAA 240

BTx623 TTAGTTTTCATCAATTATCCAAAACCATACTAGAACTTTCAATCTCATCCTTTTTGGTAA 240

**** *******************************************************

Della TTGATGACAACCCATTTACAAAGAGTTTGAACAAAATTTTCTTGGATTTCATGTGTGCTT 300

Razinieh TTGATGACAACCCATTTACAAAGAGTTTGAACAAAATTTTCTTGGATTTCATGTGTGCTT 300

BTx623 TTGATGACAACCCATTTACAAAGAGTTTGAACAAAATTTTCTTGGATTTCATGTGTGCTT 300

************************************************************

Della GCCCAAATATTTTACCATGTGTAAAGGTTATGGACAAGTTCCATGAACTTAAATTGGTAG 360

Razinieh GCCCAAATATTTTACCATGTGTAAAGGTTATGGACAAGTTCCATGAACTTAAATTGGTAG 360

BTx623 CCCCAAATATTTTACCATGTGTAAAGGTTATGGACAAGTTCCATGAACTTAAATTGGTAG 360

***********************************************************

Della CAATTACTCCTCCTACATATGTGCTAAGAGTTTAGATTTGAAAGCCTACACATATGTTTG 420

Razinieh CAATTACTCCTCCTACATATGTGCTAAGAGTTTAGATTTGAAAGCCTACACATATGTTTG 420

BTx623 CAATTACTCCTCCTACATATGTGCTAAGAGTTTAGATTTGAAAGCCTACACATATGTTTG 420

************************************************************

Della AATATGAAATATAGGAGTCAATTTCTATCAAATAATGCTAAGGTGTAAAAAATAGACCTT 480

Razinieh AATATGAAATATAGGAGTCAATTTCTATCAAATAATGCTAAGGTGTAAAAAATAGACCTT 480

BTx623 AATATGAAATATAGGAGTCAATTTCTATCAAATAATGCTAAGGTGTAAAAAATAGACCTT 480

************************************************************

ABRE, ABA-responsive element

Della TGAAACGTGACACCAATACCTTGGTATGTTTTTTTCTCTCATAGTAAATCAACATAAACA 540

Razinieh TGAAACGTGACACCAATACCTTGGTATGTTTTTTTCTCTCATAGTAAATCAACATAAACA 540

BTx623 TGAAACGTGACACCAATACCTTGGTATGTTTTTTTCTCTCATAGTAAATCAACATAAACA 540

************************************************************

ABRE, ABA-responsive element

Della TCACCACAAGCTAAAATTCAGCGAAAGGAACGTGGATGTGCTGGAACTCAGCTGCAGGTG 600

Razinieh TCACCACAAGCTAAAATTCAGCGAAAGGAACGTGGATGTGCTGGAACTCAGCTGCAGGTG 600

BTx623 TCACCACAAGCTAAAATTCAGCGAAAGGAACGTGGATGTGCTGGAACTCAGCTGCAGGTG 600

************************************************************

Della CTGCGCCTGCGCTGATCTTTTCCTTATTGATGCTCTGCAgAGGGCATAGCTGGCGCTAAT 660

Razinieh CTGCGCCTGCGCTGATCTTTTCCTTATTGATGCTCTGCAGAGGGCATAGCTGGCGCTAAT 660

BTx623 CTGCGCCTGCGCTGATCTTTTCCTTATTGATGCTCTGCAGAGGGCATAGCTGGCGCTAAT 660

************************************************************

Della CTTTGTGGACGAGGGGATCGCTTTTGCAGTTGGGTTGGGCTTTGTTTAGATCCAAAAACT 720

Razinieh CTTTGTGGACGAGGGGATCGCTTTTGCAGTTGGGTTGGGCTTTGTTTAGATCCAAAAACT 720

BTx623 CTTTGTGGACGAGGGGATCGCTTTTGCAGTTGGGTTGGGCTTTGTTTAGATCCAAAAACT 720

************************************************************

Della TTTTGGATTTTGATACTGTAGCACTTTCGTTTTTATTTGACAAAGCAACTAGACTTAAAA 780

Razinieh TTTTAGATTTTGATACTGTAGCACTTTCGTTTTTATTTGACAAAGCAACTAGACTTAAAA 780

BTx623 TTTTGGATTTCGATACTGTAGCACTTTCGTTTTTATTTGACAAAGCAACTAGACTTAAAA 780

**** ***** *************************************************

Della GATTCGTCTCGTGATTTACAAGTAAACTGTGCAATTAGTTATCTTTTTTTTATCTATATT 840

Razinieh GATTCGTCTCGTGATTTACAAGTAAACTGTGCAATTAGTTATCTTTTTTTTATCTATATT 840

BTx623 GATTCGTCTCGTGATTTATAAGTAAACTGTGCAATTAGTTATCTTT-TTTTATCTATATT 839

****************** *************************** *************

Della TAATGTTCCATGCATGTGCCGCAAGATTTGATGTGATGGGAAATCTTGTAAAGTTTTGGG 900

Razinieh TAATGTTCCATGCATGTGCCGCAAGATTTGATGTGATGGGAAATCTTGTAAAGTTTTGGG 900

BTx623 TAATGTTCCATGCATGTGCCGCAAGATTTGATGTGATGGGAAATCTTGTAAAGTTTTGGG 899

************************************************************

Della TTTTTGGGTGTATGTAAACAAAGCCTTAGGGTCGGTAGCAAGTAAAATGAACTGACCGTG 960

Razinieh TTTTTGGGTGTATGTAAACAAAGCCTTAGGGTCGGTAGCAAGTAAAATGAACTGACCGTG 960

BTx623 TTTTTGGGTGTATGTAAACAAAGCCTTAGGGTCGGTAGCAATTAAAATGAACTGACCGTG 959

***************************************** ******************

Della GAGCGGAACTCGAGAACTTGGCTGATTGGTAGTGCTAGTGCAAAGCTACCTACTACTGCG 1020

Razinieh GAGCGGAACTCGAGAACTTGGCTGATTGGTAGTGCTAGTGCAAAGCTACCTACTACTGCG 1020

BTx623 GAGCCGAACTCGAGAACTTGGCTGATTGGTAGTGCTAGTGCAAAGCTACCTACTACTGCG 1019

**** *******************************************************

Della CACGTAAAAGGTTGCACTTGAGTCGTGAATTGCAGACAGCACAATGTGAATGAACATGCA 1080

Razinieh CACGTAAAAGGTTGCACTTGAGTCGTGAATTGCAGACAGCACAATGTGAATGAACATGCA 1080

BTx623 CACGTAAAAGGTTGCACTTGAGTCGTGAATTGCAGACAGCACAATGTGAATGAACATGCA 1079

************************************************************

Della ACAACAATAATAAGCAGCTGTAGTTGTGTGTCATGTACCCAAAGATAGGTACGGAGTAGT 1140

Razinieh ACAACAATAATAAGCAGCTGTAGTTGTGTGTCATGTACCCAAAGATAGGTACGGAGTAGT 1140

BTx623 ACAACAATAATAAGCAGCTGTAGTTGTGTGTCATGTACCCAAAGATAGGTACGGAGTAGT 1139

************************************************************

Dehydration responsive element (DRE)

Della TTTATTCCTAGTCCCATGACACTGACCGACAGAAGCTCTCACCAAACAAACAATGGGAGT 1200

Razinieh TTTATTCCTAGTCCCATGACACTGACCGACAGAAGCTCTCACCAAACAAACAATGGGAGT 1200

BTx623 TTTATTCCTAGTCCCATGACACTGACCGACAGAAGCTCTCACCAAACAAACAATGGGAGT 1199

************************************************************

Della CAGCTGTCTGAGTTTCTTTGGTTGTGTGTAATCCATCAGTGATGATGACTCCGGgAGCGC 1260

Razinieh CAGCTGTCTGAGTTTCTTTGGTTGTGTGTAATCCATCAGTGATGATGACTCCGGGAGCGC 1260

BTx623 CAGCTGTCTGAGTTTCTTTGGTTGTGTGTAATCCATCCGTGATGATGACTCCGGGAGCGC 1259

************************************* **********************

Della caATCGCGCACCACAACGCACTTCACATCGGAGGAACTGGAAAaCTGGGTTGGGTCGAGT 1320

Razinieh CAATCGCGCACCACAACGCACTTCACATCGGAGGAACTGGAAAACTGGGTTGGGTCGAGT 1320

BTx623 CAATCGCGCACCACAACGCCCTTCACATCGGAGGAACTGGAAAACTGGGTTGGGTCGAGT 1319

******************* ****************************************

Della CGCGAATGGCAGTGCGCCAGgCAATGGGAGTCAACAACAGGACGCACACGCACGGAGTGA 1380

Razinieh CGCGAATGGCAGTGCGCCAGGCAATGGGAGTCAACAACAGGACGCACACGCACGGAatGa 1380

BTx623 CGCAAATGGCAGTGCGCCAGGCAATGGGAGTCAACAACAGGACGCACACGCACGGAGTGA 1379

*** **************************************************** ***

Della caGgCGTCGGCGCGAGGCTGCTGCTGGaaTGCTGGCtGgcGCATCACGCGCAATTTCCCT 1440

Razinieh CAGGCGTCGGCGCGAGGCTGCTGCTGGAGTGCTGGCTGGCGCATCACGCGCAATTTCCCT 1440

BTx623 CAGGCGTCGGCGCGAGGCTGCTGCTGGAGTGCTGGCTGGCGCATCACGCGCAATTTCCCT 1439

**************************** *******************************

Della CCCATTtCATTTTTCTACTCCTCCCTCCCCTCCCCTcccctttCctTccCcttcAcaggC 1500

Razinieh CCCATTTCATTTTTCTACTCCTCCCTCCCC-----TCCCCTTTCCTTcCCCTTCACCGGC 1495

BTx623 CCCATTTCATTTTTCTACTCCTCCCTCCCCTCCCCTCCCCTTTCCTTCCCCTTCACCGGC 1499

****************************** ********************* ***

Della AggCacGCagGGCAcGGCAcGgCacggCAGGcTTcTctcCctGATctgccctgccgagcC 1560

Razinieh AGGCACGCACGGCACGGCACGGCACGGCAGGCTTCTCTCCCTGATCTGCCCTGCCCTGCC 1555

BTx623 AGGCACGCACGGCACGGCACGGCACGGCAGGCTTCTCTCCCTGATCTGCCCTGCCCTGCC 1559

********* ********************************************* ***

Dehydration responsive element (DRE)

Della ctgcctGGAGTGGAGTCTCgCtcTcctggtAcTcCAgcccgcCcTGCCCTcaGAccccGA 1620

Razinieh CTGCCTGGAGTGGAGTCTCGCTCTCCTCGTACTCCAGCCCGCCCTGCCCTCCGACCCCGA 1615

BTx623 CTGCCTGGAGTGGAGTCTCGCTCTCCTCGTACTCCAGCCCGCCCTGCCCTCCGACCCCGA 1619

*************************** *********************** ********

Dehydration responsive element (DRE)

Della ccGACCACCGCTGCCGCAGCGACACcTACACGCCCGCCCGCcGcGgcTGAGCCTCAAC-c 1679

Razinieh CCGACCACCGCTGCCGCAGCGACACcTACACGCCCGCCcGCCGCGGCTGAGCCTCAACcg 1675

BTx623 CCGACCACCGCTGCCGCAGCGACACCTACACGCCCGCCCGCCGCGGCTGAGCCTCAAC-C 1678

**********************************************************

Della cCAGATCtCAcGCCGCGGCCACCAGATatgcGgcgcgcccgCCAtGGACGCcGGCaCCGG 1739

Razinieh cCAGATCTCACGCCGCGGCCACCAGATCTGCGGCGCGCCCGCCATGGACGCCGGCACCGG 1735

BTx623 CCAGATCTCACGCCGCGGCCACCAGATCTGCGGCGCGCCCGCCATGGACGCCGGCACCGG 1738

*************************** ********************************

Della GGGCGGcGGgcCAACGgCCATCCGCgtcccctAccacCACctccgagAcGGagaGaaGgA 1799

Razinieh GGGCGGCGGgCCAACGGCCATCCGCGTGCcCTACCGCCACCTcCGCGACGCcGAGATGGA 1795

BTx623 GGGCGGCGGGCCAACGGCCATCCGCGTGCCCTACCGCCACCTCCGCGACGCCGAGATGGA 1798

*************************** ******* ********* **** **** ***

MeJA-responsive element

Della ggtcgTcAgCctC 1812

Razinieh GctcGTCAGCcTC 1808

BTx623 GCTCGTCAGCCTC 1811

* ***********

**SUT6 promoter**

Della GTCcCTTccAGCTCCACTgTCACTGCTGGAGCCAAATCCAACTTGATGTAAGTGATGAGT 60

Razinieh GTcagTTCTAGCTCCACTGTCaCTGCTGGAGCCAAATCCAACTTGATGTAAGTGATGAGT 60

BTx623 GTCAGTTCTAGCTCCACTGTCACTGCTGGAGCCAAATCCAACTTGATGTAAGTGATGAGT 60

*** *** ***************************************************

Della GATCTGTGATAGTGATGATATGGATTTATACttGgTACGCCCACACTCATTTTCTAACAA 120

Razinieh GATCTGTGATAGTGATGATATGGATTTATACTTGG-ACGCCCACACTCATTTTCTAACAA 119

BTx623 GATCTGTGATAGTGATGATATGGATTTATACTTGG-ACGCCCACACTCATTTTCTAACAA 119

*********************************** ************************

Della GGCGTCTAGGAGAGGATAGGTTAGGGTGATTACAGGTCCAAGGTCCCATAAAAGGTGTTA 180

Razinieh GGCGTCTAGGAGAGGATAGGTTAGGGTGATTACAGGTCCAAGGTCCCATAAAAGGTGTTA 179

BTx623 GGCGTCTAGGAGAGGATAGGTTAGGGTGATTACAGGTCCAAGGTCCCATAAAAGGTGTTA 179

************************************************************

Della GTAAAAAAAGTAGCAACATGTCTTGATGATTTagAGGTCTTGTCGATGTGATATTGAGGT 240

Razinieh GtAAAaAAAGTAGCAACATGTCTTGATGATTTAGAGGTCTTGTCGATGTGATATTGAGGT 239

BTx623 GTAAAAAAAGTAGCAACATGTCTTGATGATTTAGAGGTCTTGTCGATGTGATATTGAGGT 239

************************************************************

Della GAGATTAGATCCCCGAATAGTAAGGGtTTGATGGCCTAACACTTCAAGATAGGTATTGCC 300

Razinieh GAGATTAGATCCCCGAATAGTAAGGGTTTGATGGCCTAACACTTCAAGATAGGTATTGCC 299

BTx623 GAGATTAGATCCCCGAATAGTAAGGGTTTGATGGCCTAACACTTCAAGATAGGTATTGCC 299

************************************************************

Della CATAGTGTATCCATGTTCCACACAAGTTGTAGAATCACATCTTTTCGAAGCaaAACATAT 360

Razinieh CATAGTGTATCCATGTTCCACACAAGTTGTAGAATCACATCTTTTCGAAGCAAAACATAT 359

BTx623 CATAGTGTATCCATGTTCCACACAAGTTGTAGAATCACATCTTTTCGAAGCAAAACATAT 359

************************************************************

Della TTAAAATAGGGAGTAGTTTGAATAAATTGGATATTTCgCTGAATTAtTAtcaAAAGAAAA 420

Razinieh TTAAAATAGGGAGTAGTTTGAATAAACTGGATATTTCGCTGAATTATTATGAAAAGAAAA 419

BTx623 TTAAAATAGGGAGTAGTTTGAATAAATTGGATATTTCGCTGAATTATTATGAAAAGAAAA 419

************************** *********************** *********

Della GGACATTGCACTTgGAATCCTGGCCCTTCTTCAAAATtgAaatcggAaaCTtgAAgATTC 480

Razinieh GGACATTGCACTTGGAATCCTGGCCCTTCTTCAAAATTGACGCAGGAAAGCTTGAAGATT 479

BTx623 GGACATTGCACTTGGAATCCTGGCCCTTCTTCAAAATTGACGCAGGAAAGCTTGAAGATT 479

**************************************** ***** * * *

Della GCGACGTGCGAGGCTTGCaTgTagcTTTTCTTCTTaaTGAAatACACATGaaGTCcGTtA 540

Razinieh CGGACGTGCGAGGCTTGCAGA---GTTTTCTTCTTAATGAAATACACATAAAGTCGTGTT 536

BTx623 CGGACGTGCGAGGCTTGCAGAGTT---TTCTTCTTAATGAAATACACATGAAGTCG--TG 534

***************** ********************** ***** *

Della tgGGAGAAAAAAAatCGAAtATGCTCCAAGCGAGGAGGATCTCTGGAGATATGGGGGCTG 600

Razinieh ATAGAAAAAAAAAATCAAATATGCTCCAAGCGAGGAGGATCTCTAGAGATATGGGGGCTG 596

BTx623 TTATAGAAAAAAAATCGAATATGCTCCAAGCGAGGAGGATCTCTGGAGATATGGGGGCTG 594

* ********** *************************** ***************

Della GCAGGAGGAGGTAGAGATCCGGCAAGCGGGCAGTGTGGAGCTCCTGCGCCGTGGACTTGC 660

Razinieh GCAGGAGGAGGTAGAGATCCGGCAAGCGGGCAGCGTGGAGCTCTTGCGCTGTGGACTTGC 656

BTx623 GCAGGAGGAGGTAGAGATCCGGCAAGCGGGCAGTGTGGAGCTCCTGCGCCGTGGACTTGC 654

********************************* ********* ***** **********

Della CCGCCGCCAGTGGATCAAGGCTATAGGGGAGGAGGCGGCCGAGCCAAGGGAGGTGTTGTG 720

Razinieh CCGCCGCCAGTGGATCAAGGCTATAAGGGAGGAGGCGGCCGAGCCAAGGAAGGTGTTGTG 716

BTx623 CCGCCGCCAGTGGATCAAGGCTATAGGGGAGGAGGCGGCCGAGCCAAGGGAGGTGTTGTG 714

************************* *********************** **********

Della GAGGGAGGCAGCaaTAGGGATGGTGCGGCACCGCATAGGAGGGAGAAGGGGCTGATCGAG 780

Razinieh GAGGGAGGCAGCGATAGGGATGGTGCGACACCACATAGGAGGGAGAAGGGGCTGATCGAG 776

BTx623 GAGGGAGGCAGCAATAGGGATGGTGCGGCACCGCATAGGAGGGAGAAGGGGCTGATCGAG 774

************ ************** **** ***************************

Della TGCATGGGAGGGGCAAGCAGGGAACACGACTCTGCGTTTTCCAAATCGCGCT-GAaCTCC 839

Razinieh TGCATGGGAGGGGCAAGCAGGGAACACGACTCTGCGTTTTCCAAATCGCAGAGCAACTCC 836

BTx623 TGCATGGGAGGGGCAAGCAGGGAACACGACTCTGCGTTTTCCAAATCGCGCT-GAACTCC 833

************************************************* ******

Della CATTTCGTGCACCGTTGCGTTTTCCAACGCCAACGCATGTTCGTGTTCACCCCACCGTCG 899

Razinieh CATTTCGGGCACCGTTGCGTTTTCCAACGCCAACGCATGTTCGTGTTCACCCCACCGTCG 896

BTx623 CATTTCGTGCACCGTTGCGTTTTCCAACGCCAACGCATGTTCGTGTTCACCCCACCGTCG 893

******* ****************************************************

Della CGACCCGTTCAAAGGCGGAAACAAACATACATGCCCTGCCAGTCTCAATGGAGTTTCATG 959

Razinieh CGACCCGTTCAAAGGCGGAAACAAACATACATGCCCTGCCAGTCTCAATGGAGTTTCATG 956

BTx623 CGACCCGTTCAAAGGCGGAAACAAACATACATGCCCTGCCAGTCTCAATGGAGTTTCATG 953

************************************************************

Della AAAGTTTCATGCACATTAAATATGCTGATGTGGCGCTGTAGTAATGAAGAGAGAGATGAT 1019

Razinieh AGAGTTTCATACACATTAAATATGCTGATGTGGCGCTGTAGTAATGAAGAGAGAGATGAT 1016

BTx623 AAAGTTTCATGCACATTAAATATGCTGATGTGGCGCTGTAGTAATGAAGAGAGAGATGAT 1013

* ******** *************************************************

Della AAGAGTTTCATGAAAGTAGAGAGAGTTTCATCCGCATAAAACTTCAATGCAATGTTTATA 1079

Razinieh AATAGTTTCATGGGAGTAGAGAGAGTTTCATCCGCATAAAACTTCAATGCAACGTTTATA 1076

BTx623 AAGAGTTTCATGAAAGTAGAGAGAGTTTCATCCGCATAAAACTTCAATGCAATGTTTATA 1073

** ********* ************************************** *******

Della AAATATGGATGTGTTGAAAACTGGGTCACGAAACTTTCATTGAAAATGATCTTAGTTTAT 1139

Razinieh AAATATAGATGTGTTGAAAACTGGGTCATGAAACTTCCATTGAAAATGATCTTAGTTTAT 1136

BTx623 AAATATGGATGTGTTGAAAACTGGGTCACGAAACTTTCATTGAAAATGATCTTAGTTTAT 1133

****** ********************* ******* ***********************

Della GTGAGTTTCACTTCCACCACACAATATAGAGAGGGAAAACATGTGACAGCTGCGCTGTGA 1199

Razinieh GTGAGTTTCACTTCCACCACACAATATAGAGAGAGGAAACATGTGACAGCTGCGCTGTGA 1196

BTx623 GTGAGTTTCACTTCCACCACACAATATAGAGAGGGAAAACATGTGACAGCTGCGCTGTGA 1193

********************************* * ************************

MeJA-responsive element (DRE)

Della TGCGCGTCACTTTTGCTTCTGTTATCATTCTCACCCGGTTGCTGCGGCATGCGGATAAGC 1259

Razinieh TGCGCGTCACTTTTGCTTCTGTTCTCATTCTCACCCGGTTGCTGCGGCATGCGGATAAGC 1256

BTx623 TGCGCGTCACTTTTGCTTCTGTTATCATTCTCACCCGGTTGCTGCGGCATGCGGATAAGC 1253

*********************** ************************************

Della TAGCGCTGCGGAGAAATCTAGCTGCATGCCCATTATCAAATCAAATTGAAGTTTCCTTGT 1319

Razinieh TAGCGCTGCGGAGAAATCTAGCTGCATGCCCATTATCAAATCAAATTGAAGTTTCCTTGT 1316

BTx623 TAGCGCTGCGGAGAAATCTAGCTGCATGCCCATTATCAAATCAAATTGAAGTTTCCTTGT 1313

************************************************************

Della CCATGGCTGTTGCACTATATATTATGTTGTTTGTAACGAGGCTCAGCGAAACGACGACTA 1379

Razinieh CCATGGCTGTTGCACTAT--ATTATGTTGTTTGTAACGAGGCTCAGCGAAACGACGACAA 1374

BTx623 CCATGGCTGTTGCACTATATATTATGTTGTTTGTAACGAGGCTCAGCGAAACGACGACAA 1373

****************** ************************************** *

Della GAGAGAACAACCCAACAGCTAGGCTGTGTCAAATAGGTCAACAGACCATCCCCACTGAGC 1439

Razinieh GAGAGAACAACCCAACAGCTAGGTTGTGTCAAATAGGTCAACAGACCATCCCCACTGAGC 1434

BTx623 GAGAGAACAACCCAACAGCTAGGCTGTGTCAAATAGGTCAACAGACCATCCCCACTGAGC 1433

*********************** ************************************

Della ACCACATACACTCTCCCTCTAAACTCTGATCAGAAGCGCCAATAATAATGCAAGGTCTCC 1499

Razinieh ACCACATACACTTTCCCTCTAAACTCTGATCAGAAGCGCCAATAATAATGCAAGGTCTCC 1494

BTx623 ACCACATACACTCTCCCTCTAAACTCTGATCAGAAGCGCCAATAATAATGCAAGGTCTCC 1493

************ ***********************************************

Della ATCGAAATCATTGTTCAGAATCTGTTTTGTTAATTCCTTTCCTTGAGGACCATCCAAGCA 1559

Razinieh ATCGAAATCATTGTTCAgAaTCTATTTGTTAAT-TCCTTTCCTTGAGGACCATCCAAGCA 1553

BTx623 ATCGAAATCATTGTTCAGAATCTGTTTTGTTAATTCCTTTCCTTGAGGACCATCCAAGCA 1553

*********************** *** * * **************************

Della AACA-------------------------------------------------------- 1563

Razinieh AACAATACATATATTGGAGGCGGTTTGTTAATTCCTTTCCTTGAGGACCATCCAAGCAAA 1613

BTx623 AACA-------------------------------------------------------- 1557

****

Della ATACATATATATTGGAGGCGGCTGGCTAATTCTTTTCTGTTCCTTGCTCGATCTTTGGAA 1623

Razinieh CAATACATATATTGGAGGCGGCTGGCTAATTCTTTTCTGTTCCTTGCTCGATCTTTGGAA 1673

BTx623 ATACATATATATTGGAGGCGGCTGGCTAATTCTTTTCTGTTCCTTGCTCGATCTTTGGAA 1617

* * ******************************************************

MeJA-responsive element

Della CTATGCTGGAAATTAAGCTGGCTACGCCGTCACGCTTCATTCGTCGTTCCCGCGCTCGGC 1683

Razinieh CTATGCTGGAAATTAAGCTAGCTACGCCGTCACGCTTCATTCGTCGTTCCCGCGCTCGGC 1733

BTx623 CTATGCTGGAAATTAAGCTGGCTACGCCGTCACGCTTCATTCGTCGTTCCCGCGCTCGGC 1677

******************* ****************************************

Della GCTCGTCGCTTTCCAACCCCATCCTTCCCGTCCGTGACCGTGATTATTTGCCGTGTGCTT 1743

Razinieh GCTCGTCGCTTTCCAACCCCATCCTTCCCGTCCGTGACCGTGATTATTTGCCGTGTGCTT 1793

BTx623 GCTCGTCGCTTTCCAACCCCATCCTTCCCGTCCGTGACCGTGATTATTTGCCGTGTGCTT 1737

************************************************************

Della GTTTCGTTCGTTCCTGCTCCTCTCACTCCTCGCACCTTGCGCCGCCGCAGCCCGCAGCAG 1803

Razinieh GTTTCGTTCGTTCCTGCTCCTCTCACTCCTCGCACCTTGCGCCGCCGCAGCCCGCAGCAG 1853

BTx623 GTTTCGTTCGTTCCTGCTCCTCTCACTCCTCGCACCTTGCGCCGCCGCAGCCCGCAGCAG 1797

************************************************************

MeJA-responsive element

Della CCATGGACGACGGTGACGTCGGCGAGGAGGACGCCAACaAGCAGCGCCTCGAGCGGGCCA 1863

Razinieh CCATGGACGACGGTGACGTCGGCGAGGAGGACGCCAACAAGCAGCGCCTCGAGCGGGCCA 1913

BTx623 CCATGGACGACGGTGACGTCGGCGAGGAGGACGCCAACAAGCAGCGCCTCGAGCGGGCCA 1857

************************************************************

Della CCATGAACCTGGAGCGCGGCGTCGTCGCCGGCGAGAAGGgcGACGGCAGTGGCGGGAATG 1923

Razinieh CCATGAACCTGGAGCGCGGCGTCGTCGCCGGCGAGAAGGGCGACGGCAGTGGCGGGAATG 1973

BTx623 CCATGAACCTGGAGCGCGGCGTCGTCGCCGGCGAGAAGGGCGACGGCAGTGGCGGGAATG 1917

************************************************************

Della CGAGCCGGAAgCCGCCGATAGGCATCgTCCGGCTCTTCTTgGCCTGCATGGTCtg 1978

Razinieh CGAGCCGGAAGCCGCCGATAGGCATCGTCCGGCTCTTCTTGGCCTGCATGGTCTC 2028

BTx623 CGAGCCGGAAGCCGCCGATAGGCATCGTCCGGCTCTTCTTGGCCTGCATGGTCTC 1972

******************************************************
